# Supplementary material for: Toward global integration of biodiversity big data: a harmonized metabarcode data generation module for terrestrial arthropods
Source: Gigascience. 2022 Jul 19;11:giac065. doi: 10.1093/gigascience/giac065 (PMC9295367; doi:10.1093/gigascience/giac065)
Supplement: giac065_GIGA-D-21-00420_Revision_1 [file giac065_giga-d-21-00420_revision_1.pdf]

GigaScience

Toward global integration of biodiversity big data: a harmonised metabarcode data generation module for terrestrial arthropods

--Manuscript Draft--

Manuscript Number:	GIGA-D-21-00420R1
Full Title:	Toward global integration of biodiversity big data: a harmonised metabarcode data generation module for terrestrial arthropods
Article Type:	Review
Funding Information:	Horizon 2020 (810729) — Dr Anna Papadopoulou<br>"la Caixa" Foundation (LCF/BQ/PR21/11840006) — Dr Paula Arribas
Abstract:	Metazoan metabarcoding is emerging as an essential strategy for inventorying biodiversity, with diverse projects currently generating massive quantities of community-level data. The potential for integrating across such datasets offers new opportunities to better understand biodiversity and how it might respond to global change. However, large-scale syntheses may be compromised if metabarcoding workflows differ from each other. There are ongoing efforts to improve standardisation for the reporting of inventory data. However, harmonisation at the stage of generating metabarcode data has yet to be addressed. A modular framework for harmonised data generation offers a pathway to navigate the complex structure of terrestrial metazoan biodiversity. Here, through our collective expertise as practitioners, method developers and researchers leading metabarcoding initiatives to inventory terrestrial biodiversity, we seek to initiate a harmonised framework for metabarcode data generation, with a terrestrial arthropod module. We develop an initial set of submodules covering the five main steps of metabarcode data generation: (i) sample acquisition, (ii) sample processing, (iii) DNA extraction, (iv) PCR amplification, library preparation and sequencing, and (v) DNA sequence and metadata deposition, providing a backbone for a terrestrial arthropod module. To achieve this, we (i) identified key points for harmonisation; (ii) reviewed the current state of the art; and (iii) distilled existing knowledge within submodules, thus promoting best practice by providing guidelines and recommendations to reduce the universe of methodological options. We advocate the adoption and further development of the terrestrial arthropod module. We further encourage the development of modules for other biodiversity fractions, as an essential step toward large-scale biodiversity synthesis through harmonization.
Corresponding Author:	Paula Arribas<br>Instituto de Productos Naturales y Agrobiología<br>San Cristobal de la Laguna, Canarias SPAIN
Corresponding Author Secondary Information:	
Corresponding Author's Institution:	Instituto de Productos Naturales y Agrobiología
Corresponding Author's Secondary Institution:	
First Author:	Paula Arribas
First Author Secondary Information:	
Order of Authors:	Paula Arribas
	Carmelo Andújar
	Kristine Bohmann
	Jeremy R deWaard
	Evan P Economo
	Vasco Elbrecht

Powered by Editorial Manager® and ProduXion Manager® from Aries Systems Corporation

	Stefan Geisen
	Marta Goberna
	Henrik Krehenwinkel
	Vojtech Novotny
	Lucie Zinger
	Thomas J Creedy
	Emmanouil Meramveliotakis
	Víctor Noguerales
	Isaac Overcast
	Hélène Morlon
	Anna Papadopoulou
	Alfried P Vogler
	Brent C Emerson
**Order of Authors Secondary Information:**	
**Response to Reviewers:**	Dear Editor,<br>Many thanks for your comments with respect to our manuscript entitled “Toward global integration of biodiversity big data: a harmonised metabarcode data generation module for terrestrial arthropods”. We are pleased that the editor and reviewers viewed the work timely and of general interest to the broad scientific audience of GigaScience. We found the comments helpful and interesting and have carefully read through and acted on all reviewer suggestions (all changes highlighted in the newly submitted version), and explain our responses, point-by-point, in this letter (text in blue preceded by ' >> ').<br>Many thanks for your time,<br>We look forward to hearing from you.<br>Yours sincerely,<br>Paula Arribas & Brent C. Emerson (on behalf of co-authors)<br><br><br>-----------------------------<br>Editor comments:<br><br>Dear Dr Arribas,<br>Your manuscript "Toward global integration of biodiversity big data: a harmonised metabarcode data generation module for terrestrial arthropods" (Review Article, GIGA-D-21-00420) has been assessed by our reviewers. Based on these reports, and my own assessment as Editor, I am pleased to inform you that it is potentially acceptable for publication in GigaScience, once you have carried out some essential revisions suggested by our reviewers. Their reports are below. Please note, reviewer #2 requested a document with line numbers during review, and I added those to your word document and re-uploaded it to Editorial Manager - please download the version with line numbers from EM to see which lines the reviewer refers to.<br>>> Thank you very much for the overall positive evaluation of our manuscript. We have revised the text to incorporate the suggestions of the reviewers. We are very grateful for the time that they have dedicated to reviewing our work, and their suggestions have improved clarity and provide for a more polished manuscript. Please see below for details.<br><br><br>Reviewer reports:<br>Reviewer #1: Comments to Authors (please, see pdf for a better format).<br><br>I found the manuscript "Toward global integration of biodiversity big data: a harmonised metabarcode data generation module for terrestrial arthropods" very interesting and useful.<br>I think that a review of the current metabarcoding methods and techniques is timely

and the provided structure in modules seems to work well, adding a lot of value to the work. The suggested module framework can be very valuable, especially considering it leaves options for customization.
I personally think that the literature review is quite complete, and the information reported give the reader a good picture of the topic.
>> Thank you for your positive evaluation, and for the time you have dedicated improving the clarity of our manuscript, we appreciate it.
I have a few suggestions that I hope may help making the paper even clearer and more useful to the reader. For example, I think the structure of the paper should match the structure of the figure, with a clear subdivision in modules and submodules (i.e., chapters and subchapters). While it surely is not the ideal behaviour, we are all aware that many readers will probably skim through the article to the different modules they are interested on. A more defined chapter structure of the article will make it more useful to a wider audience.
>> We think that the limited way we integrated between the figure and text may have created some confusion, and thus we have clarified this aspect in the new version. Please see below the details in the specific comment.
The writing is clear, and the article is well-written. I hope the authors will forgive me if I spent a bit of time making (probably picky) changes to the wording, especially in the conclusion. This is only because I think the paper really has value and the conclusion will be one of the most-read parts of the article once published.
>> We don’t think this is picky at all, and appreciate the time you have invested in our work. All the suggestions have been incorporated into the new version.
I have a few main comments and some minor changes (below), but I think the article should be accepted after their corrections.
Please, add continuous line numbers when resubmitting.
>> Done.
Main comments:
Module 1: Sample acquisition.
-At page 7, the authors start with the "Sample acquisition" chapter, which is their "Module 1" in the figure. The title of this chapter should be "Module 1: Sample acquisition". Here the reader, after a first introductory paragraph where a sample is defined (see next comment), should be able to find three subsections: submodule 1.1: malaise traps, submodule 1.2: pitfall traps. This proposed structure could also help readers focus on the part they are interested in. For example, if a researcher is using only pitfall traps, they will go directly to submodule 1.2. What about submodule 1.3? It appears in the figure but not in the text. If the figure is just an example of submodules that can be added, the authors should state that.
>> Thank you for pointing out this potential source of confusion. Please note that the section “Sample acquisition” does not correspond with Module 1 in Figure 1. We have clarified in the text and figure legend that Figure 1 is a schematic representation of the modular structure proposed for building a harmonised framework for the generation of metabarcode data for different fractions of terrestrial animals (modified text, lines 136). Our manuscript proposes this framework, and develops one such module: the terrestrial arthropods module, which focused on harmonisation for this biodiversity fraction.
The rationale for the structure of the terrestrial arthropod module text is that, once the justification of the general need for this module is developed in the text, we (i) review the existing literature pertaining to the five main steps (as subsections) and based on that, (ii) propose one or several submodules per step, providing a summary table for each submodule. We have reworded the text and headings (e.g. modified text, lines 136, 149, 186) to clarify this. We agree with the reviewer that many readers will probably skim through the article to the different submodules they are interested in, so thus we provide a summary table for each proposed submodule. So, for the first step (1. Sample acquisition step) of the terrestrial arthropod module we propose the 1.1 Malaise trapping sample acquisition submodule (Table 1) and the 1.2 Pitfall trapping sample acquisition submodule (Table 2). Additional submodules can be further developed within this module (e.g. 1.3, 1.4…).
-This module start saying that it's important to have a "sample definition", and I totally agree. However, the authors do not provide one. It is true that a sample definition is strongly linked to the collection technique, but I think that we still require a sample definition, and I think the authors should be able to provide one. In my opinion, all the information necessary for a definition is in the text, it just need to be summarised. For example, a sample is composed by the arthropods, but also by their preservation, and

Powered by Editorial Manager® and ProduXion Manager® from Aries Systems Corporation

	the associated metadata. If any of these factors is missing, the sample is not fit for metabarcoding. It would be useful to know what else is really required for a sample to be defined as such. This sample definition should be after the first sentence and prior to the second one.<br>>> Please see the previous comment, and note that information regarding the sample definition for each submodule is summarized in the corresponding table for each (i.e. Table 1 for the 1.1 Malaise trapping sample acquisition submodule and Table 2 for the 1.2 Pitfall trapping sample acquisition submodule).<br>-After the first introductory aspects on definition of a sample, the authors cite the work of Montgomery and colleagues, where seven different collection methods are listed. The authors state that these methods provide "an appropriate platform from which to develop sample acquisition submodules". After reading this, I would have expected the authors to provide a submodule on EACH of these seven methods. Instead, only malaise traps and pitfall traps are presented. By doing this, the authors are either contradicting themselves and the work of Montgomery, or they are not clear on the reason they decide to report only two methods. Are the authors suggesting that, of the seven sampling techniques proposed, only malaise traps and pitfall traps are good for metabarcoding? Or are you suggesting that these two alone can provide good-enough results? Or again that, while all seven techniques are good, the authors are explaining only two? (If so, why?). In any case, this should be explained in detail.<br>>> We do not suggest that only malaise traps and pitfall traps are good for metabarcoding, rather we suggest that these two provide a useful minimum set for providing broad representation. We are perhaps not clear enough on this in our original text, and we have now sought to be clearer on this point (new text, lines 261). We discuss that the seven arthropod sampling methods proposed by the review of Montgomery et al. 2021 are a solid basis to develop submodules within the Sample Acquisition step of the terrestrial arthropod module. We then review existing arthropod metabarcoding literature, and identify malaise traps as the most relevant in terms of its (i) dominant use compared to the other sampling methods, and (i) lack of harmonisation. We then identify pitfall trapping as complementary to malaise trapping because it is directed to less dispersive ground active species (modified text, lines 260). Please note that we also further encourage the development of additional submodules within the terrestrial arthropod module (new text, lines 566). However, we consider malaise and pitfall trapping to be an appropriate minimum set.<br>-In addition to this, I would separate the sampling techniques from the metadata collection, or it could get very repetitive. In fact, independently of the collection method, the metadata information should always accompany the arthropods sample. For example, why in table 1.2 is not reported "Extreme weather events during trapping"? This is very important for pitfall traps, too. Indeed, a major rainfall could dilute the preservative or even make the trap overflow (with relative risk of losing specimens). While a very hot weather is known to cause evaporation, with the risk of drying the trap. The authors mention this in the text, but not in the table.<br>>> We agree with the reviewer that Sampling Event metadata can be repetitive across sample acquisition submodules. However, we feel strongly that it is so should be considered as an essential part of sample acquisition, and so we prefer to maintain it in each sample acquisition submodule. We agree with the reviewer the ‘Extreme weather events during trapping’ metadata is a key point also for submodule 1.2 and have incorporated the info in the corresponding Table 2.<br>-In the same table, since it is reported the solution % for ethanol, also the glycol solution % should be reported. When using glycol in pitfall traps, the percentage should be lower than 95% (ideally between 40%/80% due to the viscosity of this preservative). At a 95% concentration, glycol may be so viscous that insect are not entirely submerged when they fall in the trap.<br>>> We have clarified the concentration of the propylene glycol in Table 2.<br>Module 2: Sample processing.<br>-As for the previous module, submodule paragraphs would be very helpful.<br>>> Please see our previous comment on this.<br>-I think the authors make an interesting point on the fact that size-sorting is not as necessary as one would think when deeper sequencing depth is an option. However, I have some issues with the explanations for this statement. The authors state that "increasing sequencing depth by 3-4 fold" to a "sufficient sequencing depth", together with "reasonable size ranges" make size-sorting superfluous. All these terms, unfortunately, are extremely subjective and do not enable the reader to understand when a sufficient sequencing depth is reached. Telling the reader that they need a

Powered by Editorial Manager® and ProduXion Manager® from Aries Systems Corporation

"sufficient sequencing depth" to be able to ignore size-sorting is a tautology: it is obvious that if the sequencing depth is sufficient your work is good. In my opinion, the question readers would ask themselves is: what is a good sequencing depth in order for me to avoid size-sorting as the authors suggest? It is mentioned an increase of 3-4 fold, but that is relative to the whatever number of reads you had to start with. It would probably be useful for the reader to understand what platform the authors are referring to at this stage, but that would also require the authors to explain how many samples they would process per run. Depending on the work conducted, an increase of 3-4 folds in sequencing depth may mean the operator has to move from a MiSeq to a NovaSeq, for example. Or reduce the number of samples processed on each run (or their replicates). These factors should be considered, or at least mentioned, when suggesting that a higher sequencing depth is better than size-sorting. If the reader makes it to the end of the modules, they will notice this topic is mentioned at page 21. I think, however, that the correlation between sample processing and sequencing depth is extremely important and should be explained in this module.
>> The reviewer makes a good point, and we think the simplest way to deal with it is to remove the explicit mention of 3-4 fold. Indeed, 3-4 fold is specifically relevant to the reference being cited. We agree with the reviewer that the increase in sequencing depth will depend on project specific parameters, and thus there is no magic number. We thus make the general point that increased sequencing depth is an alternative to size sorting (modified text, lines 311). We also have reworded the text to direct the reader to the discussion of step 4 (Amplification, library preparation and sequencing step section) on the sequencing depth (new text, lines 315).
I agree that size-sorting is terribly time-consuming and therefore expensive; however, having to run your samples on two runs instead of one to get a better sequencing depth would be probably more expensive. I am not sure if it can be useful to the authors, but Piper and colleagues (GigaScience, 8, 2019, 1-22, doi: 10.1093/gigascience/giz092, which is cited as reference number 7) provide a table with the costs and Gb output for each platform. This may be useful to give a reader an idea of what a good sequencing depth can be. Or link the readers to the page 21 explanation of the average reads-per-specimen expected in each sample. Otherwise, a possibly simpler solution could be to provide the reader with a method to determine what a good sequencing depth looks like. For example, a taxa recovery graph that reaches plateau has been considered a valid and easy test to determine this (Hajibabaei et al. 2019 - PLoS One. 2019; 14(9): e0220953. doi: 10.1371/journal.pone.0220953).
>> Thank you for these relevant references. We have included them in the corresponding section (new text, lines 505).
Minor changes:
Page 5, line 4: remove "are". It should read: "by placing different fractions of terrestrial diversity at the core of each "module".
>> Done.
Page 6, first 8 lines of the "Harmonisation for the metabarcoding of terrestrial arthropods" paragraph: Compared to the rest of the introduction, this paragraph could be improved both in form and in content. It seems a few different topics have just been put together, with an isolated sentence for each, without going in depth enough and without linking the sentences to each other. I suggest the authors either rewrite this paragraph or simply list the reasons why arthropods assessment is useful (e.g., biodiversity assessment, conservation of declining species, monitoring of invasives). As per the form, the use of terms such as "overwhelming" and "tremendous" could be avoided (a bit too subjective), as it should be the repetition of the word "present" at line 2.
>> We have reworded this paragraph according to reviewer suggestions (modified text, lines 151).
Page 6, last line: remove "in". It should read: "comparable to standard methods of arthropod monitoring".
>> Done.
Page 7, line 5: Close parenthesis after the references and remove the comma.
>> Done.
Page 7, first line of "Sample acquisition": "Starting point" instead of "departure point".
>> Done.
Page 15, "DNA extraction" Chapter, line 10: The authors mention the "taxonomic content of samples" and in bracket give the definition of OTUs. This can be confusing for the reader. The taxonomic content of a sample is not necessarily defined by OTUs,

Powered by Editorial Manager® and ProduXion Manager® from Aries Systems Corporation

but could be extrapolated using ASVs (amplicon sequence variants). Since the authors are referring to a specific paper they are referencing, I suggest to change the sentence to: "When assessing the recovered taxonomic content of samples using operational taxonomic units (OTUs), intact samples performed at least comparable.
>> Changed.
Page 16: The authors suggest that 100-200 µl of DNA extraction buffer can be considered appropriate for harmonisation. This gives the impression the authors are suggesting to use only 200 units of buffer when performing the DNA extraction. In my experience, an average pitfall trap that has been in the field for a week an contains even just 2 bees and 2 beetles (very unlikely) can easily require almost 1 ml of buffer when using a non-destructive DNA extraction method. As the authors stated a few sentences earlier, this is a large volume of buffer. Then why suggesting that 100-200 µl is enough? Was this referring to the use of just 100-200 µl as a subsample to purify from the overall volume used? If so, the sentence should read something like:
"Given this consideration, typical commercial kit extraction volumes of 100-200 µl can be considered an appropriate sub-sampling volume for subsequent purification."
>> Yes, that was our point. Changed (modified text, lines 387).
Page 17, Chapter 4: gene names should be italicised. Correct to: "Cytochrome c oxidase subunit I barcode region". Please, note that "subunit I" is not part of the name and should not be italicised.
>> Done.
Page 18, Line 3: I would break the sentence in two: "The BF3 fragment (418 bp) provides better taxonomic resolution than other overlapping fragments. Furthermore, primers within this region are also unaffected by slippage, and provide maximum overlap across already published studies."
>> Done.
Page 18, Line 18: Reference is missing, check "ref".
>> Included.
Page 18, Line 20: My understanding is that the proofreading activity of a polymerase is the 3′→5′ exonuclease activity. I am not sure what the "non" refers to. I think it should read: "their proofreading activity (3′→5′ exonuclease activity)"
>> Corrected.
Page 23, "Conclusion" Line 1: No need to give both the full name and the abbreviation for wocDNA, since this was done previously. The authors can pick one.
>> Done.
Page 23, "Conclusion" Line 7: "address this issue".
>> Done.
Page 23, "Conclusion" Line 8 and 11: the use of the term "canalization", while technically correct, seems a bit odd and adds unnecessary jargon, especially considering the conclusion will be read by most readers. I would suggest changing this term.
>> Changed.
Page 23, "Conclusion" Line 13 and 14: "submodule", "modular" and "modules" in the same sentence makes it very hard to read.. A possible solution could be:
"the flexible structure we presented here seeks to broaden the applicability of a modular framework within the wocDNA metabarcoding community."
>> Replaced.
Page 23, "Conclusion" Line 18: Again, it is a bit repetitive to mention the submodule structure of the module. If it is a submodule, then it is already given that is part of the module. I would rephrase by removing "module".
>> Removed.

Reviewer #2:

The manuscript makes a well-argued case for the adoption of consistent metabarcoding data generation workflows (harmonisation) for inventorying macro-biodiversity, within a modular framework, to enable larger-scale analyses that incorporate multiple datasets - and this is clearly a good idea. To do this, the authors review the relevant literature, and based on this, provide sets of workflow recommendations, at five key data generation steps, within a proposed terrestrial arthropod metabarcoding module.
The paper is largely well written and easy to follow (apart from some parts detailed in the line-by-line comments below). The authors have done an excellent job of reviewing

Powered by Editorial Manager® and ProduXion Manager® from Aries Systems Corporation

the relevant literature, and the manuscript is packed with useful workflow recommendations for metabarcoding of terrestrial invertebrates. A particularly helpful aspect is the consideration of all data generation steps, from initial sampling through to the storage of sequence data and metadata.
One possible omission is that almost no mention is made of arthropods living below ground, which is an important component of terrestrial arthropod biodiversity, with another set of sampling methods and considerations. Given that the manuscript focuses on workflows for "terrestrial arthropods", I think it should at least be mentioned that that sampling for soil arthropod metabarcoding would be another submodule, but is not considered in this manuscript. Similarly, it might be helpful to suggest other modules that could or should be developed, within the conclusion?
>> Thank you for your assessment, and for the general point you raise in your last paragraph. We fully agree on the importance of considering soil arthropods. In this manuscript, we reviewed the literature and focused on developing two submodules that we find to have more immediate relevance, in terms of their already popular implementation (i.e. malaise traps), complementarity (i.e. pitfall trapping) and lack of harmonisation. Soil arthropods are an obvious candidate for further submodule development. We agree that it is worth suggesting different submodules within the conclusions that could or should be developed within the terrestrial arthropod module, and we explicitly mention soil arthropods as an important candidate group (new text, lines 566).
Are these modules going to exist anywhere apart from within this manuscript and subsequent manuscripts? It might be helpful to have a website that collects all these modules into one place for easy access, somewhat like the Earth microbiome project website.
>> We plan to place submodules in the iBioGen project webpage (https://www.ibiogen.eu/deliverables.html), together with this and subsequent manuscripts on this topic. Additionally, we have prepared a video explaining the details of the submodules proposed in this manuscript. This video is already available via the iBioGen webpage (see https://www.ibiogen.eu/dissemination.html). Please note, it still requires final editions to accommodate modifications resulting from this review process. Once updates have been implemented and our manuscript accepted, it will be disseminated through the social media of the iBioGen project, and the authors.
L 34: For inventorying biodiversity? For compiling biodiversity inventories?
>> Changed.
L 79: It is unclear whether "metabarcode inventory data" means the data resulting from metabarcoding analyses, or the data about metabarcoding methods/workflows?
>> Clarified.
L 89: I think "global microbial initiatives" is missing something. Global microbial diversity assessment initiatives? Also, I'm not sure "(even if data generation has been centralised)" is needed.
>> Reworded.
L 94: What are eDNA initiatives, as opposed to metabarcoding initiatives?
>> Clarified.
L 98: "one of the most heterogeneous groups in terms of body size"?
>> Done.
L 99: I think it would be clearer to use "inventorying of" (i.e. compiling an inventory), rather than "inventory". (Inventorying is used elsewhere, e.g. L 108, 166).
>> Reworded.
L 110: "calibration and so" seems unnecessary.
>> Removed.
L 111: It's unclear to me why catalysis of a GO network is the key challenge. Perhaps consistent workflows are implicit in a GO network? But consistent workflows could exist without a GO network too. Can you clarify how a GO network helps?
>> We agree with the reviewer and have reworded to clarify (modified text, lines 112).
L 119-122: Arguably, bioinformatic processing of raw sequence data into processed data is another key step (depending on whether "data" is the raw sequence data, or processed OTU/ASV data). Evidently, this is not within the scope of the manuscript, but it might be worth mentioning somewhere that post-sequencing aspects of metabarcoding workflows can also vary a lot, resulting in incomparable datasets. However, this is less problematic because one can theoretically re-process the sequence data from different studies in a consistent manner.
>> We fully agree with the reviewer on the importance of harmonisation for the bioinformatic processing of raw sequence data, and we have recently published

Powered by Editorial Manager® and ProduXion Manager® from Aries Systems Corporation

	specifically on this topic (Creedy et al. 2022 (our reference 34). We have now mentioned this aspect in the manuscript, as suggested by the reviewer (new text, lines 87).<br>L 140-142: This sentence is very confusing. "long-view" should probably be "long-term goal"; "synthetic analyses" sounds like analyses of synthetic (artificial or man-made) data; and I'm not sure what "a function of any collateral costs" means. Please rephrase.<br>>> Reworded.<br>L 144: minimal compromise, if any?<br>>> Reworded.<br>L 150: The declines of insects (plural) are now a very real and serious threat?<br>>> Corrected.<br>L 161: inventorying arthropod biodiversity?<br>>> Reworded.<br>L 162: Remove "in".<br>>> Done.<br>L 183-184: panacea? Might be better to say "no one method detecting the entire arthropod diversity within a site"<br>>> Done.<br>L 273: I'm not sure "for harmonisation" is needed here.<br>>> Removed.<br>L 321: Photographing of invertebrate samples is an excellent idea!<br>>> Thanks.<br>L 330: Would there be a benefit to trying to orient all the specimens in the same way, for potential future visual-based identifications? (probably time-consuming though).<br>>> This is ideal but very time-consuming in most types of arthropod bulk samples, that is why we did not include it.<br>L 307: "4mm sieve pooled 1:10 to 2:10" is unclear. Does it mean, the < 4mm and > 4mm fractions are pooled together at a ratio of 1:10 to 2:10? Which fraction is the higher ratio? Please clarify.<br>>> Clarified.<br>L 337: What is a SuperGO?<br>>> Within the spatially led terrestrial GO network that we propose in Arribas et al. 2021, SuperGOs are sites where molecular community data is more intensively generated at both the temporal and the genomic axes, consistent with the idea of “model ecosystems” (Davies et al., 2012, 2014). This has now been clarified in the text (new text, lines 351).<br>L 398-405: "COI-bcr" is unnecessary, only used in this paragraph. "COI barcode" is used on line 408 to mean the same thing, and is clearer. I suggest replacing "COI-bcr" on lines 401 and 405 with "COI barcode" and COI barcode region", respectively.<br>>> Done.<br>L 405-406: This sentence should be rephrased. Multiple COI-targeted primer sets … demonstrated to efficiently characterise arthropods … particularly those with certain degenerate positions?<br>>> Reworded.<br>L 407: see Figure 2 in Elbrecht et al.? Should "second half" be 3' (prime)?<br>>> Done.<br>L 408-412: Can you provide citation for claims about BF3, and for primers BF2, III_B_F, Fol-degen-rev? I think the "primers within this region…" statement should be qualified with a word such as "published" or "tested". Maximum overlap of what among already published studies? (COI regions?) Do these primers have any limitations in terms of taxonomic coverage?<br>>> We have now provided references, and specifically cite Figure 2 in Elbrecht et al., [37] for a summary of the sequence, original citation and efficiency of each primer set (modified text, lines 424).<br>L 412: Why "eDNA metabarcoding" here, but just "metabarcoding" everywhere else?<br>>> Removed.<br>L 424: Citation missing?<br>>> Included.<br>L 471: Why would that be so? (Lower cost?)<br>>> Yes, clarified.<br>Table 5: What is BC3 fragment? I'm not sure how useful it is to recommend "degenerate primers" here - presumably it is certain specific COI-targeted degenerate primers that are recommended, not degenerate primers in general, in which case

	should they be listed here?<br>>> We have added additional details to Table 5 to clarify these aspects already included in the text.<br>L 533: I'm not sure what canalisation means. Replace with harmonisation?<br>>> Replaced.<br>L 539-540: "of modules" seems repetitive (of submodules) and unnecessary.<br>>> Removed.
Additional Information:	
**Question**	**Response**
Are you submitting this manuscript to a special series or article collection?	No
**Experimental design and statistics**<br><br>Full details of the experimental design and statistical methods used should be given in the Methods section, as detailed in our Minimum Standards Reporting Checklist. Information essential to interpreting the data presented should be made available in the figure legends.<br><br>Have you included all the information requested in your manuscript?	Yes
**Resources**<br><br>A description of all resources used, including antibodies, cell lines, animals and software tools, with enough information to allow them to be uniquely identified, should be included in the Methods section. Authors are strongly encouraged to cite Research Resource Identifiers (RRIDs) for antibodies, model organisms and tools, where possible.<br><br>Have you included the information requested as detailed in our Minimum Standards Reporting Checklist?	Yes
**Availability of data and materials**<br><br>All datasets and code on which the conclusions of the paper rely must be either included in your submission or deposited in publicly available repositories	Yes

Powered by Editorial Manager® and ProduXion Manager® from Aries Systems Corporation

(where available and ethically appropriate), referencing such data using a unique identifier in the references and in the "Availability of Data and Materials" section of your manuscript.<br><br>Have you have met the above requirement as detailed in our Minimum Standards Reporting Checklist?	

Powered by Editorial Manager® and ProduXion Manager® from Aries Systems Corporation

Manuscript Click here to access/download;Manuscript;Arribas et al_For_production.docx

Toward global integration of biodiversity big data: a harmonised metabarcode data generation module for terrestrial arthropods

Paula Arribas[1*], Carmelo Andújar[1], Kristine Bohmann[2], Jeremy R. deWaard[3,4], Evan P. Economo[5], Vasco Elbrecht[6], Stefan Geisen[7], Marta Goberna[8], Henrik Krehenwinkel[9], Vojtech Novotny[10,11], Lucie Zinger[12,13], Thomas J. Creedy[14], Emmanouil Meramveliotakis[15], Víctor Noguerales[1], Isaac Overcast[12], Hélène Morlon[12], Anna Papadopoulou[15], Alfried P. Vogler[14,16], Brent C. Emerson[1]

ORCID iDs: Paula Arribas [0000-0002-0358-8271]; Carmelo Andújar [0000-0001-9759-7402]; Kristine Bohmann [0000-0001-7907-064X]; Jeremy R deWaard []; Evan P Economo []; Vasco Elbrecht [0000-0003-4672-7099]; Stefan Geisen [0000-0003-0734-727X]; Marta Goberna [0000-0001-5303-3429]; Henrik Krehenwinkel [NA]; Vojtech Novotny [0000-0001-7918-8023]; Lucie Zinger [0000-0002-3400-5825]; Thomas J Creedy [0000-0002-7611-291X]; Emmanouil Meramveliotakis []; Víctor Noguerales [0000-0003-3185-778X]; Isaac Overcast [0000-0001-8614-6892]; Hélène Morlon [0000-0002-3195-7521]; Anna Papadopoulou [0000-0002-4656-4894]; Alfried P Vogler [0000-0002-2462-3718]; Brent C Emerson [0000-0003-4067-9858]

[1] Island Ecology and Evolution Research Group, Institute of Natural Products and Agrobiology (IPNA-CSIC), San Cristóbal de la Laguna, Spain.

[2] Section for Evolutionary Genomics, Globe Institute, Faculty of Health and Medical Sciences, University of Copenhagen, Denmark.

[3] Centre for Biodiversity Genomics, University of Guelph, Guelph, Canada.

[4] School of Environmental Sciences, University of Guelph, Guelph, Canada.

[5] Biodiversity and Biocomplexity Unit, Okinawa Institute of Science and Technology Graduate University, Japan.

[6] Centre for Biodiversity Monitoring (ZBM), Zoological Research Museum Alexander Koenig, Adenauerallee 160, D-53113 Bonn, Germany.

[7] Laboratory of Nematology, Department of Plant Sciences, Wageningen University and Research, 6708PB Wageningen, The Netherlands.

[8] Department of Environment and Agronomy, INIA-CSIC, Madrid, Spain.

[9] Department of Biogeography, Trier University, Trier, Germany.
[10] Biology Centre, Czech Academy of Sciences, Institute of Entomology, 37005 Ceske Budejovice, Czech Republic.
[11] Faculty of Science, University of South Bohemia, 37005 Ceske Budejovice, Czech Republic.
[12] Institut de Biologie de l'ENS (IBENS), Département de biologie, École normale supérieure, CNRS, INSERM, Université PSL, 75005 Paris, France.
[13] Naturalis Biodiversity Center, Leiden, The Netherlands
[14] Department of Life Sciences, Natural History Museum, London, UK.
[15] Department of Biological Sciences, University of Cyprus, Nicosia, Cyprus.
[16] Department of Life Sciences, Imperial College London, London, UK.
*Corresponding author: pauarribas@ipna.csic.es. https://orcid.org/ 0000-0002-0358-8271

Abstract

Metazoan metabarcoding is emerging as an essential strategy for inventorying biodiversity, with diverse projects currently generating massive quantities of community-level data. The potential for integrating across such datasets offers new opportunities to better understand biodiversity and how it might respond to global change. However, large-scale syntheses may be compromised if metabarcoding workflows differ from each other. There are ongoing efforts to improve standardisation for the reporting of inventory data. However, harmonisation at the stage of generating metabarcode data has yet to be addressed. A modular framework for harmonised data generation offers a pathway to navigate the complex structure of terrestrial metazoan biodiversity. Here, through our collective expertise as practitioners, method developers and researchers leading metabarcoding initiatives to inventory terrestrial biodiversity, we seek to initiate a harmonised framework for metabarcode data generation, with a terrestrial arthropod module. We develop an initial set of submodules covering the five main steps of metabarcode data generation: (i) sample acquisition, (ii) sample processing, (iii) DNA extraction, (iv) PCR amplification, library preparation and sequencing, and (v) DNA sequence and metadata deposition, providing a backbone for a terrestrial arthropod module. To achieve this, we (i) identified key points for harmonisation; (ii) reviewed the current state of the art; and (iii) distilled existing knowledge within submodules, thus promoting best practice by providing guidelines and recommendations to reduce the universe of methodological options. We advocate the adoption and further development of the terrestrial arthropod module. We further encourage the development of modules for other biodiversity fractions, as an essential step toward large-scale biodiversity synthesis through harmonization.

Keywords

Metabarcoding, arthropods, harmonisation, data generation, modular structure, biodiversity inventory, biodiversity big data integration, reproducibility, comparability.

Main text

Background

DNA metabarcoding, involving PCR-coupled high-throughput sequencing (HTS) directly from bulk or environmental samples, represents the most cost-efficient approach for obtaining molecular community profiles [1,2]. Metabarcoding is increasingly being used to characterise and monitor biodiversity, and is recognised as a substantial advance leading to a step change in multiple fields of biodiversity science (e.g. [3–5]). Diverse projects, from local to global scales, are currently generating massive quantities of site-based community-level biodiversity inventory data, including hyperdiverse assemblages or groups for which classical sampling and identification is overly complicated and time-consuming. The potential for integrating across such data, from diverse sources and time series, offers new opportunities to better understand how biodiversity is structured in space and time, and the factors that regulate it. Additionally, such integration can be leveraged for better monitoring and the development of holistic biodiversity conservation strategies, in response to global change [4,6,7]. However, collective international efforts are required to achieve optimal global integration and synthesis. While integrative efforts for harmonised site-based genomic inventories exist in the microbial realm (e.g. [8–10]), such a framework has yet to be extended to non-microbial fractions of biodiversity. However, there is an emerging consensus that such integration can be achieved within a HTS framework, analogous to the Genomic Observatories (GO) concept, first proposed by Davies et al., [11,12]. If effective strategies can be developed to harmonise the data resulting from metabarcoding studies (i.e. metabarcode inventory data), these can potentially scale up to a non-centralised network within

which global patterns and trends of biodiversity can be addressed [13].

There are ongoing efforts to maximise the potential for integrating across independent biodiversity data sets through improved standardisation for the reporting of inventory data (Humboldt Core: [14]). In the case of molecular data specifically, the GEOME initiative [15,16] promotes standardisation for the reporting of taxonomic, genomic and metadata through customisable yet standard-compliant spreadsheets that capture the temporal and geospatial context of a biosample. While recommendations have been made for the harmonisation of bioinformatic processing of raw metabarcode read data from metazoan biodiversity fractions [17], harmonisation at the stage of generating such metabarcode data has yet to be addressed, and thus remains a fundamental impediment for data integration. The success of global microbial diversity assessment initiatives has pivoted on standardised metabarcoding protocols for sampling, DNA extraction, barcode amplification/enrichment and library generation and sequencing of microbial/planktonic communities (e.g. [18,19] for the Earth Microbiome Project, EMP or [20–22] for the TARA Oceans and the Ocean Sampling Day, OSD). Despite pioneering efforts to harmonise metabarcode data generation beyond microbial biodiversity fractions (e.g. see [23,24]) further efforts are required within this expanding research area.

A harmonised framework for the generation of metabarcode data for terrestrial animals

Terrestrial metazoans constitute one of the most heterogeneous groups in terms of body size across the tree of life. Metabarcoding is emerging as an important approach for the inventorying of metazoan diversity, and is increasingly being used across the fields of community ecology, evolutionary ecology, biogeography, conservation biology, and environmental management, among others. Given the rapid development of data generation in this area, the potential for downstream synthesis across independently generated data sets may be compromised if divergent strategies are being implemented. There is already concern that nuances in metabarcoding

workflows make comparisons difficult (e.g. [25–28]). Guidance for the implementation of effective and robust sampling and sample-processing approaches is both timely and essential, and will increase the potential for broader benefits to biodiversity science through harmonisation. We believe that the overarching goal of a harmonised metabarcode framework for inventorying biodiversity should be to reduce unnecessary heterogeneity in the generation of metabarcode data, thus facilitating comparability and integration among independent metabarcode data sets. The development and implementation of consistent workflows for data generation is a key step for the bottom-up growth of a GO network for global integration and synthesis within biodiversity science, while the challenge is to also allow flexibility to successfully address objectives at the individual project level.

It has previously been argued that a harmonised framework with a "modular" structure for data generation could offer a pathway to navigate through the complex structure of terrestrial metazoan biodiversity, by placing different fractions of terrestrial diversity at the core of each "module" [13]. Within such a framework, best practices and harmonised protocols for the generation of metabarcode data can be developed for different target fractions of biodiversity (e.g. terrestrial arthropods). Within individual modules, submodules serve as the fundamental building blocks that provide guidelines and recommendations for the five key steps to generate metabarcode data: (i) sample acquisition, (ii) sample processing, (iii) DNA extraction, (iv) PCR amplification, library preparation and sequencing, and (v) DNA sequence and metadata deposition. Different data generation pipelines can be configured within a module by choosing among submodule options, allowing for variable requirements of different assemblages within the module (e.g. flying, aquatic or ground arthropods within a terrestrial arthropod module), and different sample vouchering needs (e.g. destructive vs non-destructive DNA extraction). Such a modular structure provides a harmonised framework for comparability across independent studies, by reducing redundant efforts, and improving reporting and comparability, while retaining flexibility to

incorporate additional submodules as the need arises (see Figure 1, a schematic representation of the proposed modular structure).

Here, through our collective expertise as practitioners of metabarcoding, method developers and researchers leading metabarcoding initiatives to inventory terrestrial arthropod biodiversity, we seek to initiate a harmonised framework for the generation of terrestrial metazoan metabarcode data. Specifically, we aim to provide an initial set of submodules (black blocks in Fig. 1) covering the five main steps of metabarcode data generation (rows 1 to 5 in Fig.1) that constitute the backbone of a terrestrial arthropod module (red block in Fig.1). We first: (i) identify key points for harmonisation within each of the five steps; (ii) review the current state of the art within the arthropod metabarcoding literature, and then; (iii) distil existing information and knowledge within submodules, thus promoting best practice by providing guidelines and recommendations to reduce the universe of methodological options. Standardisation or harmonisation of methods will, in some contexts, lead to trade-offs against what might be considered perfect methods [29]. Such trade-offs may limit the uptake of harmonised protocols, thus compromising the discovery of unifying principles from analyses synthesising across comparable studies. Thus, rather than being overly prescriptive, we seek to propose a flexible framework that can be opted into with minimal compromise, to increase the comparative value of metabarcode data.

Harmonisation for the metabarcoding of terrestrial arthropods: the terrestrial arthropods module

There are multiple reasons why techniques for inventorying and monitoring terrestrial arthropod biodiversity are urgently needed. Firstly, arthropods comprise the majority of known animal species in terrestrial habitats. It has been estimated that there are 5.5 million insect species on Earth, most yet to be discovered, and up to 6.8 million species (range 5.9–7.8 million) for all terrestrial arthropods [30]. In addition to this high diversity, arthropods present vast trait variation,

which imposes a substantial challenge for assessing their responses to environmental change. We now face the challenge of declining arthropod abundance and richness, a very real and serious threat that society must urgently address [31,32]. Arthropods are also a key biodiversity fraction for monitoring because they include many invasive species [33], requiring comparable baseline data to study the potential susceptibility and responses of communities to invasion. DNA metabarcoding has emerged as a powerful approach for characterising complex, and in many cases largely unknown, arthropod assemblages [7,34]. In response to this, researchers from diverse disciplines are shifting from conventional inventorying of arthropod diversity to DNA metabarcoding, with evidence for exponential growth uptake [17]. Indeed, adaptations of microbial metabarcoding approaches to the macroscopic component of diversity have been heavily influenced by their application to the arthropod fauna (see [1,35] for pioneering studies). Metabarcoding of DNA extracted from bulk samples of whole organisms (whole organism community DNA, wocDNA) is: (i) the most common and straightforward metabarcoding approach to inventory arthropod biodiversity; (ii) comparable to standard methods of arthropod monitoring, and; (iii) has high potential for harmonisation [27].

Data generation practices for the metabarcoding of arthropod community samples are still in the early stages. Through the development and adoption of a standardized terrestrial arthropod data generation module, the potential for comparability across future large-scale biodiversity inventorying efforts can be optimised. There is sufficient background from which recommendations can be developed (e.g. [36–40]) to guide methodological decisions within the emerging research community. Recent global initiatives that pivot on arthropod wocDNA also provide a critical mass for developing harmonised data generation, while simultaneously highlighting the relevance and timeliness of a terrestrial arthropod module. These initiatives include the BIOSCAN initiative (https://ibol.org/programs/bioscan/) and its regional extensions such as BIOSCAN Europe (https://www.bioscaneurope.org/), BioAlfa, the Kruger Malaise

Program [41], the SITE-100 project (https://www.site100.org/), the Insect Biome Atlas Project (https://insectbiomeatlas.org), LIFEPLAN (https://www.helsinki.fi/en/projects/lifeplan), and the OKEON initiative (https://okeon.unit.oist.jp/).

Identifying key points of harmonisation for submodules within each data generation step

1. Sample acquisition step

A starting point for integration across independent biodiversity inventory efforts is a harmonised sample definition. In the case of terrestrial arthropods, sample definition is strongly linked to the sampling technique implemented. There is extensive evidence that different arthropod mass sampling techniques have differing capture efficiencies with regard to total community assemblages within which they are deployed, with no one method detecting the entire arthropod diversity within a site [42]. In this context, with the aim of standardizing insect inventorying and monitoring methods, Montgomery et al., [43] proposed seven main sampling methods with the aim of maximising data integration across insect monitoring efforts, including: (i) Malaise trapping, (ii) light trapping, (iii) pan trapping, (iv) pitfall trapping, (v) beating sheets, (vi) acoustic monitoring, and (vii) active visual surveys. These complementary sampling methods provide an appropriate platform from which to develop sample acquisition submodules, which could be implemented individually or combined for more complex sampling designs.

Most implementations of wocDNA metabarcoding to date are Malaise-trap based, at scales ranging from local to global (e.g. [44–49]). Additionally, Malaise traps are frequently deployed together with other sampling techniques to generate plot-based arthropod inventory data (e.g. [50], SITE100, ForestGEO arthropod protocol), and are the sampling strategy of the Global Malaise Trap Program/BIOSCAN initiative [44], with more than 10K samples already generated worldwide. Malaise traps [51] are primarily effective for sampling flying insects (e.g. [52]) but have gained popularity for assessing terrestrial arthropod communities (e.g. [53]), and have been

proposed as ideal for insect biomonitoring using metabarcoding [43,50]. Once installed, they require limited effort and can yield clean samples comprising almost exclusively arthropods, and in very large numbers (up to 10,000 specimens per week in some cases). Moreover, they can remain in place and yield new samples through passive sampling with low handling time, making them suitable for time-resolved monitoring. Given these considerations, Malaise traps are an obvious sampling submodule candidate.

Following the recommendations of Montgomery et al., [43], together with operational procedures adopted within the BIOSCAN initiative (https://biodiversitygenomics.net/resources/bioscan), Townes-style Malaise traps are preferred, with a 165 × 110 cm interception area being most common, and 95% ethanol as the preservation agent (see [50]) but propylene glycol (ratio of 50-100% propylene glycol, with water is frequently recommended as evaporation is negligible compared to ethanol and adequately preserves DNA [54,55]). Sampling effort has typically been delimited to one week within most metabarcoding studies, representing a compromise between maximising sampling effort and reducing potential problems with DNA degradation [38]. The Malaise trap should preferably be placed at the centre of the habitat patch to be characterised and, when possible, the trap should be positioned at a right angle to the dominant insect flight line. While submodule implementation can be restricted to a single trap, we emphasize that biological replicates (simultaneous Malaise trapping events) are desirable within the same habitat patch [56], and can provide useful information regarding sampling efficiency (see e.g. [57,58] for occupancy modelling using some means of sampling replication for insects). Similarly, temporal replication is also desirable, considering the possible variability due to changing environmental conditions for optimal arthropod activity, and species-specific idiosyncrasies. If temporal replication is not possible, trapping during maximum activity periods for flying insects is desirable. See Table 1 for a summary of key guidelines and recommendations for the 1.1 Malaise trapping sample acquisition submodule.

Recording metadata associated with sampling is also an important action for harmonisation. Our opinion converges on a minimum set of metadata attributes for each sample: (i) the geographical coordinates of the Malaise trap; (ii) the date and time interval for the sampling event, and; (iii) photo recording (ideally a 360º photo around each trap) of the habitat patch within which the Malaise trap is placed. In agreement with Montgomery et al. [43], we also recommend metadata reporting for the presence of rainfall, or extreme weather events during the trapping. Detailed characterisation of habitat and microhabitats within sampling sites would require time and resources that may limit module uptake. If needed, environmental characterisation of sampling sites can potentially be extracted from remote sensing data (see [4]). For additional information on metadata reporting, see section 5, DNA sequence and metadata sharing and storage.

Sample storage conditions, as the endpoint of the sample acquisition chain, carry implications for downstream data quality, and are thus an important focus for harmonisation. Sample storage conditions are consequential for the degradation of target DNA and/or the proliferation of non-target biomass in the sample. As such, they can strongly impact metabarcoding biodiversity profiles [59]. However, the effect of this bias on mock arthropod samples, at least for short-term storage (i.e. < 1 month), is of limited importance (see [38]). In the case of longer storage of arthropod community samples, we strongly recommend the use of >95% molecular grade ethanol as a preservative using leak-proof glass or plastic vials or jars [60], ensuring that the entire bulk sample is fully submerged before storage and then storage conditions of −20 or −80ºC. In the case of storage or transport safety constraints, propylene glycol (undiluted) can be used as an alternative to ethanol [61]. Such an approach will limit inherent biases in inventory data due to irregular DNA degradation. The storage of biological replicates is always desirable (Table 1).

Table 1. Summary of key guidelines and recommendations within the *1.1 Malaise trapping sample acquisition submodule.*

**1.1 Malaise trapping sample acquisition submodule**	
Sample definition	Townes-style Malaise trap (165 × 110 cm interception area)<br>One week per sample<br>Collecting fluid: >95% ethanol/propylene glycol<br>Centre in habitat patch location<br>Position perpendicular to natural flight corridor<br>Spatial and temporal replicates
Sampling event metadata	Geographical coordinates<br>Date and period of trapping<br>Photo recording for habitat and microhabitat<br>Extreme weather events during trapping
Sample storage	>95% molecular grade ethanol/propylene glycol<br>Fully submerged biomass<br>Storage conditions of -20º or -80ºC

While Malaise trapping is notably efficient for aerially active arthropods, species with low mobility are less likely to be sampled (e.g. [62]). In this context, pitfall trapping offers a complementary passive sampling technique for ground active arthropods, and thus we consider it to be an appropriate candidate for the development of a complementary sampling submodule. The joint implementation of malaise and pitfall trapping represents an appropriate compromise to limit the diversity of sampling techniques implemented, while seeking to capture a broad representation of arthropod biodiversity. Pitfall traps [63] are containers buried in the ground with their rim at surface level to capture ground-dwelling (epigeic) insects. Pitfall traps are the most effective method for sampling ground active arthropods, and are an established and popular monitoring technique (e.g. the US National Ecological Observatory Network [NEON], [54]; the UK Environmental Change Network, [64]). Pitfall and Malaise traps are highly complementary, sampling largely non-overlapping fractions of arthropod assemblages with reduced additional

effort, and they have already been jointly applied in several wocDNA metabarcoding studies (e.g. [48]).

Guidelines for standardising pitfall trapping, based on a review of the existing literature [65], have recommended plastic cups with 11 cm diameter and 9-11 cm depth, and a roof raised 1.5 cm above the trap entrance. The number of individuals sampled per trap can be limited, and as such, composite samples from multiple pitfall traps can be used to increase the sampling effort. There is some controversy over how far apart traps should be placed to be considered as independent samples (e.g. [66,67]). We suggest that the NEON protocol [54] provides a suitable framework for harmonisation, within which a composite sample is generated using four pitfall traps arranged at the corners of a square with sides of 25 m. While submodule implementation can be restricted to a single composite sample (four pitfall traps), biological replicates are desirable (e.g. [54]), and can be achieved by allowing several metres between replicate traps within each corner. Sampling effort is defined by the trapping interval and varies across studies, typically ranging from three days to four weeks (e.g. [48,54,68]). One week provides an appropriate interval, and facilitates coordination with the setting and servicing of Malaise traps. Temporal replication is also desirable and if not possible, trapping should be targeted toward periods of maximum arthropod activity [54]. Propylene glycol (ratio of 50-100% propylene glycol, with water; for a total volume between 100 and 200 mL, depending upon the dilution ratio) is the most frequently recommended collecting medium, as evaporation is negligible compared to ethanol, it is odourless, and it adequately preserves DNA ([54,55], Table 2).

Similar to Malaise traps, a minimum set of metadata attributes for each pitfall composite sample should include: (i) the geographical coordinates of the trap, (ii) period of the trapping event and (iii) photo recording (ideally a 360º photo around each trap). Following Montgomery et al. [43], we also recommend metadata reporting for the presence of rainfall or extreme events during sampling. Finally, in order to minimise the degradation of target DNA and/or the proliferation of

non-target biomass in the sample during medium-long term storage, we strongly recommend the use of >95% molecular grade ethanol, or propylene glycol, as described above for Malaise trap samples). See Table 2 for key guidelines and recommendations of the 1.2 Pitfall trapping sample acquisition submodule.

Table 2. Summary of key guidelines and recommendations within the *1.2 Pitfall trapping sample acquisition submodule.*

**1.2 Pitfall trapping sample acquisition submodule**	
Sample definition	Plastic cups with diameter: 11 cm, depth: 9-11 cm, and a roof raised 1.5 cm<br>Composite sample (four pitfall traps, placed at the corners of a square with sides of 25 m).<br>One week per sample<br>Collecting fluid: propylene glycol<br>Spatial and temporal replicates
Sampling event metadata	Geographical coordinates<br>Date and period of trapping<br>Photo recording for habitat and microhabitat<br>Extreme weather events during trapping
Sample storage	>95% mol grade ethanol/50%-95% propylene glycol<br>Fully submerged biomass<br>Storage conditions of -20º or -80ºC

2. Sample processing step

In contrast to microbial or environmental DNA (eDNA) approaches, where samples can be directly processed for DNA extraction, the macroscopic nature of arthropod community samples has led to a broad range of sample processing protocols, among which size sorting is the most common. Size sorting is often used because larger specimens tend to release more DNA and may dominate the total sequence count in metabarcoding data [69]. Thus, sorting invertebrates into multiple size classes and then pooling the digested tissue according to DNA concentration, abundance or richness in each class has become common practice (e.g. [1,70,71], and size sorting has revealed

improved efficiency in the detection of low biomass species (e.g. [40,70]). However, increasing sequencing depth can also increase taxon recovery to comparable levels without size sorting [72]. More generally, it has been suggested that with sufficient sequencing depth and within reasonable size ranges, species recovery is not skewed by variable biomass of species, and that a size sorting step need not be carried out [71]. Please see the section '4 Amplification, library preparation and sequencing step' for details on sequencing depth. In addition to the fact that handling time for size sorting places high logistical constraints for large-scale studies, size sorting procedures also reduce comparability across independent initiatives if not fully harmonised. Given these considerations, we consider size sorting to be unnecessary for a harmonised approach, but if incorporated it should be of limited complexity (e.g. wet sieving into two size fractions, 4 mm sieve pooled 1:10 to 2:10 (> 4 mm : < 4 mm), [72]) and properly reported. Removing any form of biomass sorting/sample picking steps will also improve cost-effectiveness and facilitate broad implementation for biomonitoring [27].

Biomass and abundance information is often fundamental for biodiversity analysis, including the global assessment of arthropod decline (see [73]). However, deriving abundance information from metabarcode data remains a challenge, primarily due to inherent biases during PCR amplification, but also because of variation in gene copy number, organelle number, and technical aspects of workflows for sampling, laboratory procedures, sequencing and bioinformatic processing [5,69,74]. Given these considerations, we consider that an arthropod community sample processing submodule should emphasise the importance of (i) providing a wet weight measurement for each sample and (ii) generating arthropod community sample photographs. Wet mass measurement can be used as a surrogate for sample biomass. It can be easily obtained from samples after filtering off excess ethanol using a nylon filtration fabric that retains smaller specimens (e.g. 20 µm filters).

Photographic recording is not a commonly reported practice, but looking forward we think it is very likely that the integration of quantitative morphological and molecular approaches will

be an important area of interest and development [75]. There is potential for image-based specimen identification involving machine learning tools to be applied as an external validation of molecular-based diversity estimations, particularly for arthropod groups with limited cryptic variation between species [75–77]. While obtaining high-quality images of arthropod community samples may be time-consuming, we recommend, as a minimum, that such images should be taken at high-resolution using a conventional stereoscope equipped with a built-in microscope camera or an external single lens reflex (SLR) camera with macro lens, over a white background (ideally submerged under ethanol in a plastic tray), and minimising the overlap among individuals to provide a physical record of the sample. Vouchering selected specimens may be considered unnecessary when well-parameterized reference libraries are available (e.g. [78]), but is otherwise an important consideration for future taxonomic assignment of metabarcoding reads and for completing reference barcode databases (e.g. following BOLD guidelines, see [50,79]). Vouchering also provides a resource for potential parallel efforts to generate high-throughput specimen-based genomic resources (i.e. partial or complete genomes, microbiomes, diet) for sites of special interest (SuperGOs, [13], i.e. sites where molecular community data is intensively generated at both the temporal and the genomic axes, consistent with the idea of 'model ecosystems'). Vouchered barcode sequences are also of particular relevance for bioinformatic processing of metabarcode reads. It has been demonstrated that such sequences are fundamental for efficient and validated filtering of nuclear copies of mitochondrial sequences, and that they control for taxonomically inflated estimates of community composition [80]. While sample processing is not the most problematic step for cross-contamination, contamination issues have been reported (e.g. [81]), and at least basic equipment cleaning between samples is required. See Table 3 for key guidelines and recommendations of the arthropod community sample processing submodule.

Table 3. Summary of key guidelines and recommendations within the *2.1 Arthropod community sample processing submodule*.

**2.1 Arthropod community sample processing submodule**	
Sample wet mass weight	20 µm nylon filtration fabric
Sample photography	White background<br>Ethanol submerged (white tray)<br>Photographic scale
Size sorting	Minimise size sorting<br>4 mm sieve
Vouchering specimens	Random or directed selection of specimens for being individually DNA extracted and barcoded

3. DNA extraction step

A fundamental consideration for harmonising wocDNA extraction concerns whether a pre-extraction homogenisation-grinding step (thus implying destruction of the specimens within an arthropod community sample) is needed. Such a step can facilitate homogeneous digestion across specimens, and reduce digestion volumes. It is often achieved through manual grinding in a mortar after freezing in liquid nitrogen, or grinding in ethanol, or mechanical bead beating. Non-destructive extraction protocols have been developed for unsorted arthropod samples to maintain exoskeletal integrity (e.g. [61,70,82]. Using mock arthropod community samples generated from material collected in Malaise traps, Nielsen et al., [82] found that homogenised samples yielded more DNA, but generally produced more inconsistent results when compared to non-destructive extraction. When assessing the recovered taxonomic content of samples using operational taxonomic units (OTUs), intact samples performed at least comparable to, if not better than, homogenised samples. Thus, considering that efficiency seems to be comparable, avoiding a homogenisation step will: (i) reduce potential heterogeneity among studies; (ii) reduce processing time; (iii) reduce contamination risk, and; (iv) maintain a physical archive accessible for future

developments in image classification using deep learning for the extraction of additional data, such as abundances (see 2. Sample processing). Given these considerations, non-destructive DNA extraction should be a core feature of the arthropod community sample DNA extraction submodule. When necessary (e.g. soil arthropods where a large fraction have hard exoskeletons, see [83]), semi-destructive or destructive extraction submodules will need to be developed. Non-destructive DNA extractions require large volumes of digestion buffer to extract wocDNA. Nielsen et al. [82] have demonstrated that OTU diversity estimates are not influenced by the (sub)volume of digestion buffer that is subsequently purified. Given this consideration, typical commercial kit extraction volumes of 100-200 μl can be considered an appropriate sub-sampling volume for subsequent purification.

A broad range of DNA extraction protocols are being applied to wocDNA metabarcoding. It remains unclear how different extraction methods might impact downstream results, as there is contrasting evidence on its importance based on eDNA approaches [19,84]. Manual (column-based) and robotic (bead-based) implementations of the Qiagen DNeasy Blood & Tissue kit and homologous kits have been widely used for extracting wocDNA from terrestrial invertebrates [34]. There is little evidence for PCR inhibitor issues for DNA extracts from arthropod community samples (but see [85]), and if they occur they can be appropriately accounted for through dilution of DNA extracts before PCR amplification (see next section). Given these considerations, simple and efficient kit-based protocols that allow sample extraction at scale (e.g. Qiagen DNeasy Blood & Tissue, and analogous kits, see [86]) provide an appropriate basis for harmonisation. Negative controls and technical replicates are fundamental for quality control and can be used to filter out artefactual sequences [87], and as such their incorporation in the extraction step will also facilitate validation and integration of data across studies.

Biobanking of DNA from environmental samples has been strongly advocated for long-term biomonitoring [88]. Biobanking of DNA ensures opportunities for re-analysis of past data

sets with future technologies, an important consideration given high method turnover and associated comparability issues. Aliquots of purified wocDNA are suitable for archiving, ideally using low-DNA binding tubes and freezers of -80 °C or colder, but if this option is unavailable, storage at -20 ºC in non-defrosting freezers provides an adequate alternative. Several museums are already offering this service with affordable pricing (e.g. Smithsonian & Canadian museum in Ottawa). See Table 4 for key guidelines and recommendations for the arthropod community sample DNA extraction submodule.

Table 4. Summary of key guidelines and recommendations proposed within the *3.1 Arthropod community sample DNA extraction submodule*.

**3.1 Arthropod community sample DNA extraction submodule**	
Digestion	No physical homogenisation step<br>High volumes of digestion buffer<br>Long digestion (shaking)
Purification	200µl of digestion buffer<br>Qiagen DNeasy Blood & Tissue type<br>Negative controls and technical extraction replicates
Purified DNA storage	Biobanking of DNA aliquots<br>-80º, -20º non-defrosting freezers

4. Amplification, library preparation and sequencing step

There is a clear trend toward the use of the *Cytochrome c oxidase* subunit I barcode region (COI barcode) for wocDNA metabarcoding of arthropods (e.g. [37,40,83,89–92]). This can be largely attributed to: (i) the good performance of different COI primers for arthropod community samples; (ii) the availability of large COI barcode reference databases; (iii) sufficient variation to typically allow taxonomic assignment at the species level, and; (iv) the potential to identify and remove sequencing errors and spurious sequence assemblies by bioinformatic processing based on the predicted variation in protein- coding regions and the limited expected length variation within the

COI barcode [89]. Multiple primer sets have been demonstrated to efficiently characterise arthropod community samples, particularly those incorporating degenerate nucleotide positions (i.e. positions that allow for the binding of more than one nucleotide) (see Figure 2 in Elbrecht et al., [37]), with a trend toward using the second half (3′) of the COI barcode for metabarcoding studies (e.g. [40,93]). The BF3 fragment (418 bp) provides better taxonomic resolution than other overlapping fragments. Furthermore, primers within this region are also unaffected by slippage, and provide maximum overlap across already published studies [37]. Given these considerations, choosing primers of demonstrated efficiency within the BF3 region (BF3 + BF2 or III_B_F + Fol-degen-rev, among others (see [37])), or that overlap substantially with it, offer high potential for harmonising across independent studies.

PCR conditions are strongly dependent on selected primers, but also on sample composition and polymerase used. Ideally, PCR annealing temperatures and cycle numbers should be qPCR-optimized [94]. However, in the absence of such optimization, steps can be taken to reduce unneeded variability across studies. The number of PCR cycles should be maintained at or below 30 cycles if possible, to limit the formation of intra-sample chimeras ([95], reviewed in [5]). Serial dilution is a beneficial strategy, as DNA concentration from arthropod community samples, together with PCR inhibitors can be high, potential inhibitors can be effectively diluted out (e.g., [96]). Comparisons of polymerase performance for metabarcoding [97] has revealed that polymerase choice impacts read abundance, but not occurrence. Among six commercially available polymerases tested, Qiagen Multiplex Master Mix has been shown to provide the most accurate estimates of relative abundance, but also generated the highest error rate [97]. While high-fidelity DNA polymerases can reduce PCR error rates [97,98], their proofreading activity (3′→5′ exonuclease activity) can increase the rate of chimera formation [99,100]. PCR volume does not appear to be an important consideration for harmonisation as it has been reported that it does not

influence downstream results, but provides opportunity for cost savings via PCR miniaturisation (lower cost from reduced quantities of reaction components, [101], Table 5).

Performing PCR replicates and pooling for library preparation or sequencing is a well-established standard in the metabarcoding literature, particularly for arthropod community samples, with strong recommendations for a minimum pooling of three PCR replicates [102,103]. The use of multiple PCR replicates per sample to be individually sequenced (technical replication) is less common, but their importance has been highlighted. Together with PCR negative controls, technical PCR replicates can provide important quality control for the removal of PCR and sequencing artefacts [87,94,104]. Thus, negative controls and technical replication within individual sequencing runs should be considered essential practice to identify potential biases and errors from (i) cross-contamination, (ii) tag-jumping events [105] and (iii) false-negative detection. Given the high potential for cross contamination within the PCR step, rigorous measures should be taken to minimise this risk (e.g. using filter tips, robotic platforms for plate aliquoting). Cross-contamination can be detected and filtered out by including technical replicates, together with positive and negative controls randomly distributed among different plates to bioinformatically curate data, reducing problems associated with tag switching and/or cross-contamination [106]. These should be included in the laboratory and sequencing workflow (e.g. [107]). An important measure that enables one to filter out potential contamination during data processing is to use different nucleotide tag and/or library index combinations for individual PCR replicates within samples, as this will allow for restrictive sequence processing across each replicate [87,104]. Similarly, the number of reads assigned to a given tag/library index combination that were not used in the study can provide an estimation of the contamination rate, and thus a minimum OTU relative abundance that should be considered as reliable [108]. Mock communities have been investigated as positive controls for estimating recovery bias, and the use of synthetic/exogenous internal standards has also been explored to estimate absolute abundance from metabarcode data

[10,109,110]. In the context of harmonisation across studies, universal positive controls harbour much potential for inter-calibration. This has yet to be developed and tested, but could be the basis for further improvement within this submodule.

Library preparation involves the addition of sample-specific nucleotide identifiers to amplicons and nucleotide tails for sequencing, for which there is considerable heterogeneity in the arthropod wocDNA metabarcoding literature. In their recent review, Bohmann et al., [106] identified and reviewed three main approaches to achieve sample-specific labelling and library preparation in metabarcoding studies. These include: (i) a one-step PCR approach in which sample DNA extracts are amplified, tagged and built into sequence libraries in a single PCR reaction with fusion primers, then pooled and sequenced; (ii) a two-step PCR, in which sample DNA extracts are PCR-amplified with two primer sets: a first PCR with metabarcoding primers carrying the 5' sequence overhangs and no nucleotide tags, and a second PCR using sequence overhangs, allowing the amplicons to be indexed (i5 and i7 indexes), and; (iii) a tagged PCR approach, in which DNA extracts are PCR amplified with metabarcoding primers that carry 5' nucleotide tags, individually tagged PCR products are then pooled, and PCR-based or ligation-based library preparation is performed for pools of 5' tagged amplicons.

All three labelling strategies have been used for arthropod wocDNA metabarcoding (e.g. [70,94,111]). The two-step approach, which is based on the Illumina 16S rRNA protocol, originally developed for microbiome studies, appears to be more commonly used. Tests comparing consistency and taxon detection efficiency between one step and two step PCR protocols (in this case implementing TrueSeq Nano over first untagged PCR) using mock arthropod samples reveal better performance with the two step protocol [26]. Ligation-based tagged PCR library preparations have been advocated, to avoid false assignment of sequences to samples by tag jumping [94,112], a recognised problem within the PCR-based tagged approach [105,106]. However, no study has yet compared performance between two-step and ligation-based tagged

PCR. Between these two, the two-step approach is the more frequently used for arthropod metabarcoding, and thus provides a suitable approach to minimise heterogeneity across studies (Table 5).

The sequencing depth needed to recover all taxa is strongly dependent on the diversity and complexity of a given sample. A sequencing depth of 60,000 ± 55,000 reads per amplicon per sample is commonly reported [113]. Increasing sequencing depth can increase the detection rate of low-abundance taxa and reduce the impacts of differential processing protocols on perceived diversity [40]. However, increased sequencing depth increases the cost by sample (see Table 2 in Piper et al., [7] for a summary of the costs (2019) and Gb output for each platform), and inherently increases the detection of artefactual sequences, requiring additional procedures for their removal [5,80,104]. Distinguishing between sufficient or insufficient sequencing depth can be controlled for by evaluating replicability [40], or by taxa recovery graphs on mock or composition controlled communities of comparable nature [114]. The choice of sequencing platform also has potential to generate variation among data sets. This variation appears to be limited across currently popular platforms, such as Illumina MiSeq, Ion Torrent PGM and Ion Torrent S5 [40]. However, as future sequencing platforms may present greater variation, it is important to report such details (e.g. sequencing platform, read length). See Table 5 for key guidelines and recommendations for the arthropod community sample DNA amplification, library preparation and sequencing submodule.

Table 5. Summary of key guidelines and recommendations proposed within the *4.1 Arthropod community sample DNA amplification, library preparation and sequencing submodule*.

**4.1 Arthropod community sample DNA amplification, library preparation and sequencing submodule**	
Target DNA fragments and primers	COI locus<br>Second half (3′) of the COI barcode fragment<br>Degenerate primers (see Elbrech et al. 2019)

PCR conditions	Minimize number of PCR cycles<br>Dilution of DNA extract<br>Non-proofreading Taq<br>PCR replicates (3), ideally individually labelled<br>Negative controls<br>Technical PCR replication<br>Cross-contamination control practices
Library preparation	Two-step protocol

5. Metadata and DNA sequence sharing and storage step

Metadata associated with the different steps of generating metabarcode data should be reported with DNA sequence data to enhance long term reuse value (see [115]). The GEOME (Genomic Observatories Metadatabase) initiative [15,16] offers a very useful platform, facilitating findable, accessible, interoperable and reusable data archival practices (i.e. FAIR principles). Interoperability is central to GEOME, as metadata follow controlled vocabularies consistent with DarwinCore and MIxS standards [116,117] and new records on GEOME are incorporated into the Global Biodiversity Information Facility, GBIF (https://www.gbif.org/). A customizable but standard-compliant single spreadsheet for metainformation, including: (i) the reference to the submodules implemented within each data acquisition steps (e.g. 1.2 sample acquisition submodule, 2.1 sample processing submodule, etc), and; (ii) all key information highlighted within each of the submodules, will facilitate downstream comparison among data sets. The metadata spreadsheet for the terrestrial arthropod module (GEOME spreadsheet) can be additionally included as supplementary publication material.

Finally, GEOME also facilitates DNA data sharing through the deposition of raw genetic data to the Sequence Read Archive (SRA, www.ncbi.nlm.nih.gov/sra), while maintaining persistent links to standard compliant metadata held in the GEOME database. SRA is thus an ideal platform for the storage of demultiplexed HTS files. Given the continuous development and improvement of bioinformatic tools for HTS data analysis, public archiving of raw DNA data is

important to facilitate future synthetic analysis across historical data sets. See Table 6 for key guidelines and recommendations of the arthropod community sample metadata and DNA sequence sharing and storage submodule.

Table 6. Summary of key guidelines and recommendations proposed within the *5.1 Arthropod community sample Metadata and DNA sequence sharing and storage submodule*.

**5.1 Arthropod community sample Metadata and DNA sequence sharing and storage submodule**	
Metadata	GeOME metadata submission<br>GeOME spreadsheet with the key information of the modules performed
DNA sequences	Raw data<br>SRA

Conclusions

Whole organism community DNA metabarcoding is emerging as a powerful tool to characterise and compare arthropod communities, from the scale of local community composition through to global comparative analyses. For this potential to be fully realised, comparability across data sets generated by independent research groups is a fundamental prerequisite. There are several challenges to achieve this. First, as is the case for many new fields, early development has led to different strategies and tools, among which some will facilitate data comparability, while others will not. Here we have addressed this issue by suggesting a modular framework that seeks to reduce redundant efforts and improve comparability across studies by harmonisation of common practice across different research initiatives, where that practice demonstrates utility. We have illustrated this framework with recommendations for a module for the characterisation of terrestrial arthropods. A second challenge is that canalisation of different practises to optimise comparability at the community level may, inadvertently, limit flexibility at the scale of individual studies. While

this is to some extent unavoidable, the flexible structure we presented here seeks to broaden the applicability of a modular framework within the wocDNA metabarcoding community. Finally, unless appropriate data and metadata are provided for a given wocDNA metabarcode study, the opportunities for integrative analyses across historical data sets are likely to be limited. We address this challenge by advocating good reporting practice, and highlight that the submodule structure provides a framework for the incorporation of new advances as they emerge within the field of metabarcoding. We advocate the adoption and development of the terrestrial arthropod module that we propose here, as an important step toward harmonisation of metabarcode data. We further encourage the development of additional submodules for the terrestrial arthropod module (e.g. soil mesoarthropod sample acquisition, pan trapping for pollinator sample acquisition), as well as modules for other biodiversity fractions that are appropriate targets for wocDNA metabarcoding.

Declarations:

Data Availability

Not applicable

Competing interests

A.P.V. is a co- founder and scientific advisor of NatureMetrics, a private company providing commercial services in DNA- based monitoring. The authors declare that they have no other conflicts of interest.

Funding

The working group “Toward harmonisation for the generation of metabarcoding data: Soil Biodiversity and Terrestrial Arthropod modules” held in November 2020 (online) was organized by the iBioGen project, which has received funding from the European Union’s Horizon 2020 research and innovation programme under grant agreement No. 810729. PA was funded through a Junior Leader Fellowship (LCF/BQ/PR21/11840006) by “la Caixa” Foundation (ID 100010434)

and the European Union's Horizon 2020 research and innovation program under the Marie Skłodowska-Curie grant agreement No847648.

Authors' contributions

P.A. and B.C.E. conceptualized the manuscript. All authors contributed to the ideas and discussion of this review. P.A. and B.C.E. coordinated the working group meetings and led the writing with contributions from all authors. All authors read and approved the final manuscript.

Acknowledgements

Not applicable

Figure legends

Figure 1. A harmonised framework with a "modular" structure for metazoan metabarcoding. Schematic representation of the modular structure proposed for building a harmonised framework for the generation of metabarcode data for different fractions of terrestrial animals. Different fractions of terrestrial animal diversity are at the core of each "module" (red rectangle, e.g. the terrestrial arthropods module) and within such a framework, best practices and harmonised protocols are developed as submodules (black blocks). Submodules within each module serve as the fundamental building blocks that provide guidelines and recommendations for the five, well-defined steps for generating metabarcode data (left panel, rows 1 to 5). Within this framework, tailored data generation pipelines can be configured within a module, drawn from the set of alternative submodules.

References

1. Ji Y, Ashton L, Pedley SM, Edwards DP, Tang Y, Nakamura A, et al.. Reliable, verifiable and efficient monitoring of biodiversity via metabarcoding. *Ecol Lett*. 2013; doi: 10.1111/ele.12162.

2. Porter TM, Hajibabaei M. Scaling up: A guide to high-throughput genomic approaches for biodiversity analysis. *Mol Ecol*. 2018; doi: 10.1111/mec.14478.

3. Bohan DA, Vacher C, Tamaddoni-Nezhad A, Raybould A, Dumbrell AJ, Woodward G. Next-Generation Global Biomonitoring: Large-scale, Automated Reconstruction of Ecological Networks. *Trends Ecol Evol*. 2017; doi: 10.1016/j.tree.2017.03.001.

4. Bush A, Sollmann R, Wilting A, Bohmann K, Cole B, Balzter H, et al.. Connecting Earth observation to high-throughput biodiversity data. *Nat Ecol Evol*. Macmillan Publishers Limited; 2017; doi: 10.1038/s41559-017-0176.

5. Taberlet P, Bonin A, Zinger L, Coissac E. Environmental DNA for biodiversity research and monitoring. Environ. DNA Biodivers. Res. Monit. Oxford: Oxford University Press;

6. Bush A, Catullo R, Mokany K, Harwood T, Hoskins AJ, Ferrier S. Incorporating existing thermal tolerance into projections of compositional turnover under climate change. *Glob Ecol Biogeogr*. 2019; doi: 10.1111/geb.12898.

7. Piper AM, Batovska J, Cogan NOI, Weiss J, Cunningham JP, Rodoni BC, et al.. Prospects and challenges of implementing DNA metabarcoding for high-throughput insect surveillance. *Gigascience*. Oxford University Press; 2019; doi: 10.1093/gigascience/giz092.

8. Gilbert JA, Jansson JK, Knight R. The Earth Microbiome project: Successes and aspirations. *BMC Biol*. 2014; doi: 10.1186/s12915-014-0069-1.

9. Gilbert JA, Meyer F, Antonopoulos D, Balaji P, Brown CT, Brown CT, et al.. Meeting Report: The Terabase Metagenomics Workshop and the Vision of an Earth Microbiome Project. *Stand Genomic Sci*. 2010; doi: 10.4056/sigs.1433550.

10. Ovaskainen O, Abrego N, Somervuo P, Palorinne I, Hardwick B, Pitkänen JM, et al.. Monitoring Fungal Communities With the Global Spore Sampling Project. *Front Ecol Evol*. 2020; doi: 10.3389/fevo.2019.00511.

11. Davies N, Meyer C, Gilbert JA, Amaral-Zettler L, Deck J, Bicak M, et al.. A call for an

international network of genomic observatories (GOs). *Gigascience*. 2012; doi: 10.1186/2047-217X -1-5.

12. Davies N, Field D, Amaral-Zettler L, Clark MS, Deck J, Drummond A, et al.. The founding charter of the Genomic Observatories Network. *Gigascience*. 2014; doi: 10.1186/2047-217X-3-2.

13. Arribas P, Andújar C, Bidartondo MI, Bohmann K, Coissac E, Creer S, et al.. Connecting high-throughput biodiversity inventories – opportunities for a site-based genomic framework for global integration and synthesis. *Mol Ecol*. 30:1120–352021;

14. Guralnick R, Walls R, Jetz W. Humboldt Core – toward a standardized capture of biological inventories for biodiversity monitoring, modeling and assessment. *Ecography (Cop)*. 2018; doi: 10.1111/ecog.02942.

15. Deck J, Gaither MR, Ewing R, Bird CE, Davies N, Meyer C, et al.. The Genomic Observatories Metadatabase (GeOMe): A new repository for field and sampling event metadata associated with genetic samples. *PLoS Biol*. 2017; doi: 10.1371/journal.pbio.2002925.

16. Riginos C, Crandall ED, Liggins L, Gaither MR, Ewing RB, Meyer C, et al.. Building a global genomics observatory: Using GEOME (the Genomic Observatories Metadatabase) to expedite and improve deposition and retrieval of genetic data and metadata for biodiversity research. *Mol Ecol Resour*. 2020; doi: 10.1111/1755-0998.13269.

17. Creedy TJ, Andújar C, Meramveliotakis E, Noguerales V, Overcast I, Papadopoulou A, et al.. Coming of age for COI metabarcoding of whole organism community DNA: towards bioinformatic harmonisation. *Mol Ecol Resour*. 2022; doi: 10.1111/1755-0998.13502.

18. Caporaso JG, Paszkiewicz K, Field D, Knight R, Gilbert JA. The Western English Channel contains a persistent microbial seed bank. *ISME J*. Nature Publishing Group; 2012; doi: 10.1038/ismej.2011.162.

19. Marotz C, Amir A, Humphrey G, Gaffney J, Gogul G, Knight R. DNA extraction for

streamlined metagenomics of diverse environmental samples. *Biotechniques*. 2017; doi: 10.2144/000114559.

20. Alberti A. to metazoan marine plankton nucleotide sequences from the Tara Oceans expedition. *Sci Data*. 4:1700932017;

21. Gorsky G, Bourdin G, Lombard F, Pedrotti ML, Audrain S, Bin N, et al.. Expanding Tara Oceans Protocols for Underway, Ecosystemic Sampling of the Ocean-Atmosphere Interface During Tara Pacific Expedition (2016–2018). *Front Mar Sci*. 2019; doi: 10.3389/fmars.2019.00750.

22. Kopf A. The ocean sampling day consortium. *Gigascience*. 2015; doi: 10.1186/s13742-015-0065-6.

23. Dickie IA, Boyer S, Buckley HL, Duncan RP, Gardner PP, Hogg ID, et al.. Towards robust and repeatable sampling methods in eDNA based studies. *Mol Ecol Resour*. 2018; doi: 10.1111/1755-0998.12907.

24. Canonico G, Buttigieg PL, Montes E, Muller-Karger FE, Stepien C, Wright D, et al.. Global observational needs and resources for marine biodiversity. *Front Mar Sci*. 2019; doi: 10.3389/fmars.2019.00367.

25. Murray DC, Coghlan ML, Bunce M. From benchtop to desktop: Important considerations when designing amplicon sequencing workflows. *PLoS One*. 2015; doi: 10.1371/journal.pone.0124671.

26. Zizka VMA, Elbrecht V, Macher JN, Leese F. Assessing the influence of sample tagging and library preparation on DNA metabarcoding. *Mol Ecol Resour*. 2019; doi: 10.1111/1755-0998.13018.

27. Blackman RC, Mächler E, Altermatt F, Arnold A, Beja P, Boets P, et al.. Advancing the use of molecular methods for routine freshwater macroinvertebrate biomonitoring – the need for calibration experiments. *Metabarcoding and Metagenomics*. 2019; doi: 10.3897/mbmg.3.34735.

28. Zaiko A, Greenfield P, Abbott C, von Ammon U, Bilewitch J, Bunce M, et al.. Towards reproducible metabarcoding data: Lessons from an international cross-laboratory experiment. *Mol Ecol Resour*. 2021; doi: 10.1111/1755-0998.13485.

29. Philippot L, Ritz K, Pandard P, Hallin S, Martin-Laurent F. Standardisation of methods in soil microbiology: Progress and challenges. *FEMS Microbiol Ecol*. 2012; doi: 10.1111/j.1574-6941.2012.01436.x.

30. Stork NE, McBroom J, Gely C, Hamilton AJ. New approaches narrow global species estimates for beetles, insects, and terrestrial arthropods. *Proc Natl Acad Sci*. 2015; doi: 10.1073/pnas.1502408112.

31. Goulson D. The insect apocalypse, and why it matters. *Curr Biol*. Elsevier; 2019; doi: 10.1016/j.cub.2019.06.069.

32. Harvey JA, Heinen R, Armbrecht I, Basset Y, Baxter-Gilbert JH, Bezemer TM, et al.. International scientists formulate a roadmap for insect conservation and recovery. *Nat Ecol Evol*. 2020; doi: 10.1038/s41559-019-1079-8.

33. Seebens H, Blackburn TM, Dyer EE, Genovesi P, Hulme PE, Jeschke JM, et al.. Global rise in emerging alien species results from increased accessibility of new source pools. *Proc Natl Acad Sci U S A*. 2018; doi: 10.1073/pnas.1719429115.

34. Liu M, Clarke LJLJ, Baker SC, Jordan GJGJ, Burridge CPCP. A practical guide to DNA metabarcoding for entomological ecologists. *Ecol Entomol*. 2019; doi: 10.1111/een.12831.

35. Yu DW, Ji Y, Emerson BC, Wang X, Ye C, Yang C, et al.. Biodiversity soup: metabarcoding of arthropods for rapid biodiversity assessment and biomonitoring. *Methods Ecol Evol*. 2012; doi: 10.1111/j.2041-210X.2012.00198.x.

36. Andújar C, Arribas P, Gray C, Bruce C, Woodward G, Yu DW, et al.. Metabarcoding of freshwater invertebrates to detect the effects of a pesticide spill. *Mol Ecol*. 2018; doi: 10.1111/mec.14410.

37. Elbrecht V, Braukmann TW, Ivanova N V, Prosser SW, Hajibabaei M, Wright M, et al.. Validation of COI metabarcoding primers for terrestrial arthropods. *PeerJ*. PeerJ Inc.; 2019; doi: 10.7287/peerj.preprints.27801v1.

38. Krehenwinkel H, Fong M, Kennedy S, Huang EG, Noriyuki S, Cayetano L, et al.. The effect of DNA degradation bias in passive sampling devices on metabarcoding studies of arthropod communities and their associated microbiota. *PLoS One*. 2018; doi: 10.1371/journal.pone.0189188.

39. Krehenwinkel H, Kennedy SR, Rueda A, Lam A, Gillespie RG. Scaling up DNA barcoding – Primer sets for simple and cost efficient arthropod systematics by multiplex PCR and Illumina amplicon sequencing. *Methods Ecol Evol*. 2018; doi: 10.1111/2041-210X.13064.

40. Braukmann TWA, Ivanova N V., Prosser SWJ, Elbrecht V, Steinke D, Ratnasingham S, et al.. Metabarcoding a diverse arthropod mock community. *Mol Ecol Resour*. 2019; doi: 10.1111/1755-0998.13008.

41. D'Souza ML, van der Bank M, Shongwe Z, Rattray RD, Stewart R, van Rooyen J, et al.. Biodiversity baselines: Tracking insects in Kruger National Park with DNA barcodes. *Biol Conserv*. 2021; doi: 10.1016/j.biocon.2021.109034.

42. Basset Y, Springate ND, Aberlenc HP, Delvare G. A review of methods for sampling arthropods in tree canopies. In: Strok NE, Adis J, Didham RK, editors. *Canopy Arthropods*. London: Chapman and Hall;

43. Montgomery GA, Belitz MW, Guralnick RP, Tingley MW. Standards and Best Practices for Monitoring and Benchmarking Insects. *Front Ecol Evol*. 2021; doi: 10.3389/fevo.2020.579193.

44. Geiger MF, Moriniere J, Hausmann A, Haszprunar G, Wägele W, Hebert PDN, et al.. Testing the Global Malaise Trap Program - How well does the current barcode reference library identify flying insects in Germany? *Biodivers Data J*. 2016; doi: 10.3897/BDJ.4.e10671.

45. Marquina D, Esparza- Salas R, Roslin T, Ronquist F. Establishing arthropod community

composition using metabarcoding: Surprising inconsistencies between soil samples and preservative ethanol and homogenate from Malaise trap catches. *Mol Ecol Resour*. 2019; doi: 10.1111/1755-0998.13071.

46. Barsoum N, Bruce C, Forster J, Ji YQ, Yu DW. The devil is in the detail: Metabarcoding of arthropods provides a sensitive measure of biodiversity response to forest stand composition compared with surrogate measures of biodiversity. *Ecol Indic*. Elsevier; 2019; doi: 10.1016/j.ecolind.2019.01.023.

47. Ritter CD, Häggqvist S, Karlsson D, Sääksjärvi IE, Muasya AM, Nilsson RH, et al.. Biodiversity assessments in the 21st century: The potential of insect traps to complement environmental samples for estimating eukaryotic and prokaryotic diversity using high-throughput DNA metabarcoding. *Genome*. 2019; doi: 10.1139/gen-2018-0096.

48. Watts C, Dopheide A, Holdaway R, Davis C, Wood J, Thornburrow D, et al.. DNA metabarcoding as a tool for invertebrate community monitoring: a case study comparison with conventional techniques. *Austral Entomol*. 2019; doi: 10.1111/aen.12384.

49. Hausmann A, Segerer AH, Greifenstein T, Knubben J, Morinière J, Bozicevic V, et al.. Toward a standardized quantitative and qualitative insect monitoring scheme. *Ecol Evol*. 2020; doi: 10.1002/ece3.6166.

50. DeWaard JR, Levesque-Beaudin V, DeWaard SL, Ivanova N V., McKeown JTA, Miskie R, et al.. Expedited assessment of terrestrial arthropod diversity by coupling Malaise traps with DNA barcoding. *Genome*. 2019; doi: 10.1139/gen-2018-0093.

51. Malaise R. A new insect-trap. *Entomol Tidskr*. 58:148–601937;

52. Schmidt O, Schmidt S, Häuser CL, Hausmann A, Van Vu L. Using Malaise traps for collecting Lepidoptera (Insecta), with notes on the preparation of Macrolepidoptera from ethanol. *Biodivers Data J*. 2019; doi: 10.3897/BDJ.7.e32192.

53. Karlsson D, Forshage M, Holston K, Ronquist F. The data of the Swedish Malaise Trap

Project, a countrywide inventory of Sweden's insect fauna. *Biodivers Data J*. 2020; doi: 10.3897/bdj.8.e56286.

54. Hoekman D, Levan KE, Ball GE, Browne RA, Davidson RL, Erwin TL, et al.. Design for ground beetle abundance and diversity sampling within the National Ecological Observatory Network. *Ecosphere*. 2017; doi: 10.1002/ecs2.1744.

55. Nakamura S, Tamura S, Taki H, Shoda-Kagaya E. Propylene glycol: a promising preservative for insects, comparable to ethanol, from trapping to DNA analysis. *Entomol Exp Appl*. 2020; doi: 10.1111/eea.12876.

56. Steinke D, Braukmann TWA, Manerus L, Woodhouse A, Elbrecht V. Effects of Malaise trap spacing on species richness and composition of terrestrial arthropod bulk samples. *Metabarcoding and Metagenomics*. 2021; doi: 10.3897/MBMG.5.59201.

57. Szewczyk TM, McCain CM. Disentangling elevational richness: a multi-scale hierarchical Bayesian occupancy model of Colorado ant communities. *Ecography (Cop)*. 2019; doi: 10.1111/ecog.04115.

58. Powney GD, Carvell C, Edwards M, Morris RKA, Roy HE, Woodcock BA, et al.. Widespread losses of pollinating insects in Britain. *Nat Commun*. Springer US; 2019; doi: 10.1038/s41467-019-08974-9.

59. Weißbecker C, Buscot F, Wubet T. Preservation of nucleic acids by freeze-drying for next generation sequencing analyses of soil microbial communities. *J Plant Ecol*. 2017; doi: 10.1093/jpe/rtw042.

60. Marquina D, Buczek M, Ronquist F, Lukasik P. The effect of ethanol concentration on the morphological and molecular preservation of insects for biodiversity studies. *PeerJ*. 2021; doi: 10.7717/peerj.10799.

61. Martoni F, Nogarotto E, Piper AM, Mann R, Valenzuela I, Eow L, et al.. Propylene Glycol and Non-Destructive DNA Extractions Enable Preservation and Isolation of Insect and Hosted

Bacterial DNA. *Agric*. 2021; doi: 10.3390/agriculture11010077.
62. de Kerdrel GA, Andersen JC, Kennedy SR, Gillespie R, Krehenwinkel H. Rapid and cost-effective generation of single specimen multilocus barcoding data from whole arthropod communities by multiple levels of multiplexing. *Sci Rep*. 2020; doi: 10.1038/s41598-019-54927-z.
63. Hertz M. Huomioita petokuoriaisten olinpaikoista. *Luonnon Yst*. 31:218–221927;
64. Brooks DR, Bater JE, Clark SJ, Monteith DT, Andrews C, Corbett SJ, et al.. Large carabid beetle declines in a United Kingdom monitoring network increases evidence for a widespread loss in insect biodiversity. *J Appl Ecol*. 2012; doi: 10.1111/j.1365-2664.2012.02194.x.
65. Brown GR, Matthews IM. A review of extensive variation in the design of pitfall traps and a proposal for a standard pitfall trap design for monitoring ground-active arthropod biodiversity. *Ecol Evol*. 2016; doi: 10.1002/ece3.2176.
66. Ward DF, New TR, Yen AL. Effects of pitfall trap spacing on the abundance, richness and composition of invertebrate catches. *J Insect Conserv*. 2001; doi: 10.1023/A:1011317423622.
67. Hohbein RR, Conway CJ. Pitfall traps: A review of methods for estimating arthropod abundance. *Wildl Soc Bull*. 2018; doi: 10.1002/wsb.928.
68. Missa O, Basset Y, Alonso A, Miller SE, Curletti G, De Meyer M, et al.. Monitoring arthropods in a tropical landscape: Relative effects of sampling methods and habitat types on trap catches. *J Insect Conserv*. 2009; doi: 10.1007/s10841-007-9130-5.
69. Deagle BE, Thomas AC, McInnes JC, Clarke LJ, Vesterinen EJ, Clare EL, et al.. Counting with DNA in metabarcoding studies: How should we convert sequence reads to dietary data? *Mol Ecol*. 2019; doi: 10.1111/mec.14734.
70. Elbrecht V, Peinert B, Leese F. Sorting things out: Assessing effects of unequal specimen biomass on DNA metabarcoding. *Ecol Evol*. 2017; doi: 10.1002/ece3.3192.
71. Creedy TJ, Ng WS, Vogler AP. Toward accurate species-level metabarcoding of arthropod

communities from the tropical forest canopy. *Ecol Evol*. 2019; doi: 10.1002/ece3.4839.

72. Elbrecht V, Bourlat S, Hörren T, Lindner A, Mordente A, Noll N, et al.. Pooling size sorted malaise trap fractions to maximise taxon recovery with metabarcoding. *PeerJ*. 2021; doi: 10.7717/peerj.12177.

73. Cardoso P, Leather SR. Predicting a global insect apocalypse. *Insect Conserv Divers*. 2019; doi: 10.1111/icad.12367.

74. Zinger L, Bonin A, Alsos IG, Bálint M, Bik H, Boyer F, et al.. DNA metabarcoding—Need for robust experimental designs to draw sound ecological conclusions. *Mol Ecol*. 2019; doi: 10.1111/mec.15060.

75. Ärje J, Melvad C, Jeppesen MR, Madsen SA, Raitoharju J, Rasmussen MS, et al.. Automatic image-based identification and biomass estimation of invertebrates. *Methods Ecol Evol*. 2020; doi: 10.1111/2041-210X.13428.

76. Valan M, Makonyi K, Maki A, Vondráček D, Ronquist F. Automated taxonomic identification of insects with expert-level accuracy using effective feature transfer from convolutional networks. *Syst Biol*. 2019; doi: 10.1093/sysbio/syz014.

77. Valan M, Vondráček D, Ronquist F. Awakening a taxonomist’s third eye: exploring the utility of computer vision and deep learning in insect systematics. *Syst Entomol*. 2021; doi: 10.1111/syen.12492.

78. Ronquist F, Forshage M, Häggqvist S, Karlsson D, Hovmöller R, Bergsten J, et al.. Completing Linnaeus’s inventory of the Swedish insect fauna: Only 5,000 species left? *PLoS One*. 2020; doi: 10.1371/journal.pone.0228561.

79. deWaard JR, Ratnasingham S, Zakharov E V, Borisenko A V, Steinke D, Telfer AC, et al.. A reference library for the identification of Canadian invertebrates: 1.5 million DNA barcodes, voucher specimens, and genomic samples. *Sci Data*. 2019; doi: 10.1038/s41597-019-0320-2.

80. Andújar C, Creedy TJ, Arribas P, López H, Salces-Castellano A, Pérez-Delgado AJ, et al..

Validated removal of nuclear pseudogenes and sequencing artefacts from mitochondrial metabarcode data. *Mol Ecol Resour*. 2021; doi: 10.1111/1755-0998.13337.

81. Creedy TJTJ, Norman H, Tang CQCQ, Qing Chin K, Andujar C, Arribas P, et al.. A validated workflow for rapid taxonomic assignment and monitoring of a national fauna of bees (Apiformes) using high throughput DNA barcoding. *Mol Ecol Resour*. doi: 10.1111/1755-0998.13056.

82. Nielsen M, Gilbert MTP, Pape T, Bohmann K. A simplified DNA extraction protocol for unsorted bulk arthropod samples that maintains exoskeletal integrity. *Environ DNA*. 2019; doi: 10.1002/edn3.16.

83. Arribas P, Andújar C, Hopkins K, Shepherd M, Vogler AP, Andújar C, et al.. Metabarcoding and mitochondrial metagenomics of endogean arthropods to unveil the mesofauna of the soil. *Methods Ecol Evol*. 2016; doi: 10.1111/2041-210X.12557.

84. Deiner K, Walser JC, Mächler E, Altermatt F. Choice of capture and extraction methods affect detection of freshwater biodiversity from environmental DNA. *Biol Conserv*. Elsevier Ltd; 2015; doi: 10.1016/j.biocon.2014.11.018.

85. Majaneva M, Diserud OH, Eagle SHC, Hajibabaei M, Ekrem T. Choice of DNA extraction method affects DNA metabarcoding of unsorted invertebrate bulk samples. *Metabarcoding and Metagenomics*. 2018; doi: 10.3897/mbmg.2.26664.

86. Sellers GS, Di Muri C, Gómez A, Hänfling B. Mu-DNA: A modular universal DNA extraction method adaptable for a wide range of sample types. *Metabarcoding and Metagenomics*. 2018; doi: 10.3897/mbmg.2.24556.

87. Zinger L, Lionnet C, Benoiston A-S, Donald J, Mercier C, Boyer F. metabaR : an R package for the evaluation and improvement of DNA metabarcoding data quality. *Methods Ecol Evol*. 2021; doi: 10.1111/2041-210X.13552.

88. Jarman SN, Berry O, Bunce M. The value of environmental DNA biobanking for long-term

biomonitoring. *Nat Ecol Evol*. Springer US; 2018; doi: 10.1038/s41559-018-0614-3.
89. Andújar C, Arribas P, Yu DW, Vogler AP, Emerson BC. Why the COI barcode should be the community DNA metabarcode for the Metazoa. *Mol Ecol*. 2018; doi: 10.1111/mec.14844.
90. Beng KC, Tomlinson KW, Shen XH, Surget-Groba Y, Hughes AC, Corlett RT, et al.. The utility of DNA metabarcoding for studying the response of arthropod diversity and composition to land-use change in the tropics. *Sci Rep*. 2016; doi: 10.1038/srep24965.
91. Elbrecht V, Leese F, Nichols SJ. Validation and Development of COI Metabarcoding Primers for Freshwater Macroinvertebrate Bioassessment. *Front Environ Sci*. 2017; doi: 10.3389/fenvs.2017.00011.
92. Leray M, Yang JY, Meyer CP, Mills SC, Agudelo N, Ranwez V, et al.. A new versatile primer set targeting a short fragment of the mitochondrial COI region for metabarcoding metazoan diversity: application for characterizing coral reef fish gut contents. *Front Zool*. Frontiers in Zoology; 2013; doi: 10.1186/1742-9994-10-34.
93. Arribas P, Andújar C, Salces-Castellano A, Emerson BC, Vogler AP. The limited spatial scale of dispersal in soil arthropods revealed with whole-community haplotype-level metabarcoding. *Mol Ecol*. 2021; doi: https://doi.org/10.1111/mec.15591.
94. Yang C, Bohmann K, Wang X, Cai W, Wales N, Ding Z, et al.. Biodiversity Soup II: A bulk-sample metabarcoding pipeline emphasizing error reduction. *Methods Ecol Evol*. 2021; doi: 10.1111/2041-210X.13602.
95. Haas BJ, Gevers D, Earl AM, Feldgarden M, Ward D V., Giannoukos G, et al.. Chimeric 16S rRNA sequence formation and detection in Sanger and 454-pyrosequenced PCR amplicons. *Genome Res*. 2011; doi: 10.1101/gr.112730.110.
96. Elbrecht V, Vamos EE, Steinke D, Leese F. Estimating intraspecific genetic diversity from community DNA metabarcoding data. *PeerJ*. 2018; doi: 10.7717/peerj.4644.
97. Nichols R V., Vollmers C, Newsom LA, Wang Y, Heintzman PD, Leighton M, et al..

Minimizing polymerase biases in metabarcoding. *Mol Ecol Resour*. 2018; doi: 10.1111/1755-0998.12895.

98. Sze MA, Schloss PD. The Impact of DNA Polymerase and Number of Rounds of Amplification in PCR on 16S rRNA Gene Sequence Data. *mSphere*. 2019; doi: 10.1128/msphere.00163-19.

99. Ahn JH, Hong IP, Bok JI, Kim BY, Song J, Weon HY. Pyrosequencing analysis of the bacterial communities in the guts of honey bees Apis cerana and Apis mellifera in Korea. *J Microbiol*. 2012; doi: 10.1007/s12275-012-2188-0.

100. Gury J, Zinger L, Gielly L, Taberlet P, Geremia RA. Exonuclease activity of proofreading DNA polymerases is at the origin of artifacts in molecular profiling studies. *Electrophoresis*. 2008; doi: 10.1002/elps.200700667.

101. Buchner D, Beermann AJ, Leese F, Weiss M. Cooking small and large portions of “biodiversity- soup”: Miniaturized DNA metabarcoding PCRs perform as good as large-volume PCRs. *Ecol Evol*. 2021; doi: 10.1002/ece3.7753.

102. Ficetola GF, Pansu J, Bonin A, Coissac E, Giguet-Covex C, De Barba M, et al.. Replication levels, false presences and the estimation of the presence/absence from eDNA metabarcoding data. *Mol Ecol Resour*. 2015; doi: 10.1111/1755-0998.12338.

103. Dopheide A, Xie D, Buckley TR, Drummond AJ, Newcomb RD. Impacts of DNA extraction and PCR on DNA metabarcoding estimates of soil biodiversity. *Methods Ecol Evol*. 2019; doi: 10.1111/2041-210X.13086.

104. Alberdi A, Aizpurua O, Gilbert MTP, Bohmann K. Scrutinizing key steps for reliable metabarcoding of environmental samples. *Methods Ecol Evol*. 2018; doi: 10.1111/2041-210X.12849.

105. Schnell IB, Bohmann K, Gilbert MTP. Tag jumps illuminated - reducing sequence-to-sample misidentifications in metabarcoding studies. *Mol Ecol Resour*. 2015; doi: 10.1111/1755-

0998.12402.

106. Bohmann K, Elbrecht V, Carøe C, Bista I, Leese F, Bunce M, et al.. Strategies for sample labelling and library preparation in DNA metabarcoding studies. *Mol Ecol Resour*. 2021;

107. Bista I, Carvalho GR, Tang M, Walsh K, Zhou X, Hajibabaei M, et al.. Performance of amplicon and shotgun sequencing for accurate biomass estimation in invertebrate community samples. *Mol Ecol Resour*. 2018; doi: 10.1111/1755-0998.12888.

108. Esling P, Lejzerowicz F, Pawlowski J. Accurate multiplexing and filtering for high-throughput amplicon-sequencing. *Nucleic Acids Res*. 2015; doi: 10.1093/nar/gkv107.

109. Smets W, Leff JW, Bradford MA, McCulley RL, Lebeer S, Fierer N. A method for simultaneous measurement of soil bacterial abundances and community composition via 16S rRNA gene sequencing. *Soil Biol Biochem*. Elsevier Ltd; 2016; doi: 10.1016/j.soilbio.2016.02.003.

110. Ushio M, Murakami H, Masuda R, Sado T, Miya M, Sakurai S, et al.. Quantitative monitoring of multispecies fish environmental DNA using high-throughput sequencing. *Metabarcoding and Metagenomics*. 2018; doi: 10.3897/mbmg.2.23297.

111. Elbrecht V, Leese F, Rockström J, Steffen W, Noone K, Persson A, et al.. Can DNA-Based Ecosystem Assessments Quantify Species Abundance? Testing Primer Bias and Biomass—Sequence Relationships with an Innovative Metabarcoding Protocol. *PLoS One*. 2015; doi: 10.1371/journal.pone.0130324.

112. Carøe C, Bohmann K. Tagsteady: A metabarcoding library preparation protocol to avoid false assignment of sequences to samples. *Mol Ecol Resour*. 2020; doi: 10.1111/1755-0998.13227.

113. Singer GAC, Fahner NA, Barnes JG, McCarthy A, Hajibabaei M. Comprehensive biodiversity analysis via ultra-deep patterned flow cell technology: a case study of eDNA metabarcoding seawater. *Sci Rep*. 2019; doi: 10.1038/s41598-019-42455-9.

114. Hajibabaei M, Porter TM, Wright M, Rudar J. COI metabarcoding primer choice affects richness and recovery of indicator taxa in freshwater systems. *PLoS One*. 2019; doi: 10.1371/journal.pone.0220953.

115. Tedersoo L, Ramirez KS, Nilsson RH, Kaljuvee A, Kõljalg U, Abarenkov K. Standardizing metadata and taxonomic identification in metabarcoding studies. *Gigascience*. 2015; doi: 10.1186/s13742-015-0074-5.

116. Wieczorek J, Bloom D, Guralnick R, Blum S, Döring M, Giovanni R, et al.. Darwin core: An evolving community-developed biodiversity data standard. *PLoS One*. 2012; doi: 10.1371/journal.pone.0029715.

117. Yilmaz P, Kottmann R, Field D, Knight R, Cole JR, Amaral-Zettler L, et al.. Minimum information about a marker gene sequence (MIMARKS) and minimum information about any (x) sequence (MIxS) specifications. *Nat Biotechnol*. 2011; doi: 10.1038/nbt.1823.

Figure

Click here to access/download;Figure;Figure1_final_version.eps

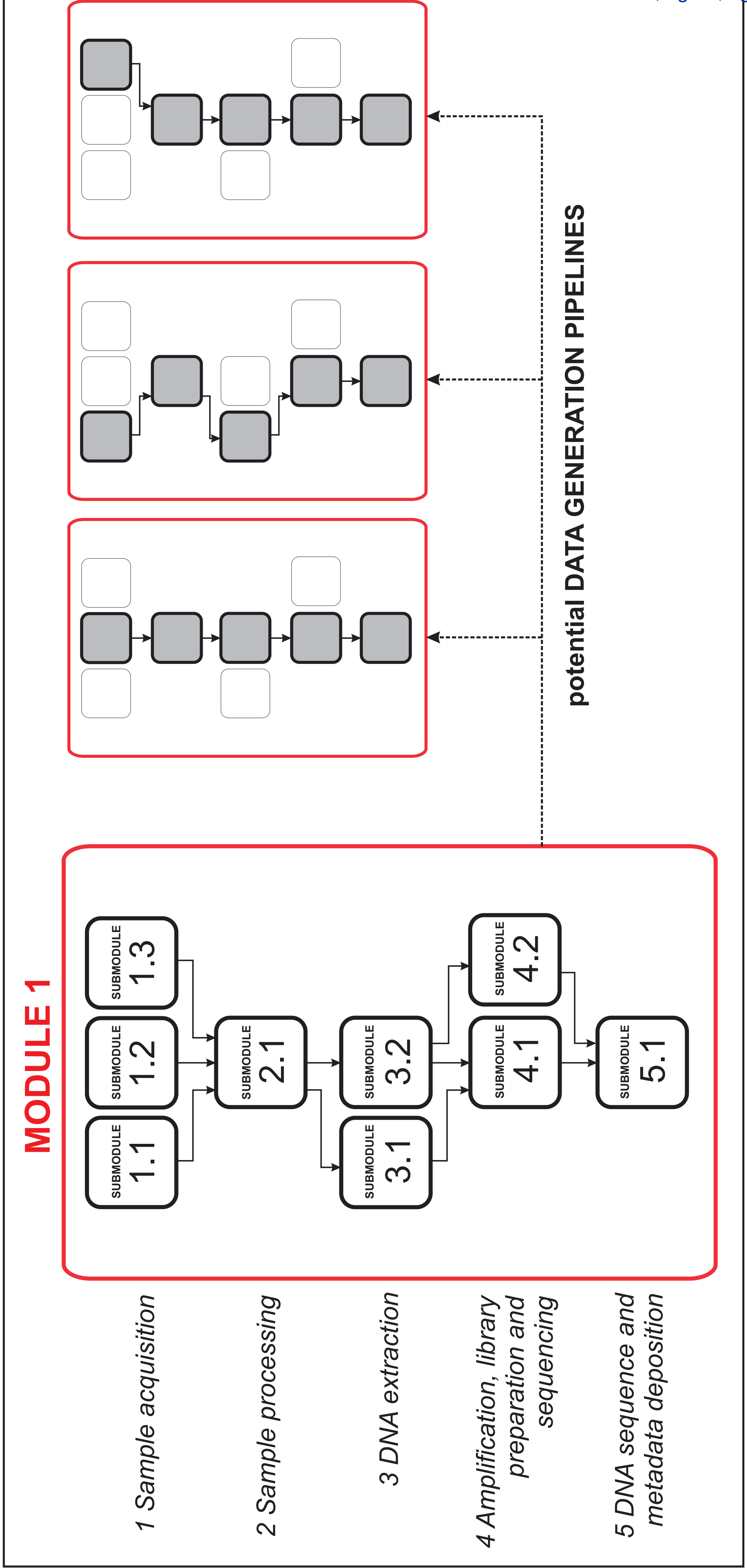

Personal Cover
Click here to access/download;Personal Cover;Arribas_et_al_cover_letter_to_submit_final_version.docx

Dear Editor,

Many thanks for your comments with respect to our manuscript entitled *"Toward global integration of biodiversity big data: a harmonised metabarcode data generation module for terrestrial arthropods"*. We are pleased that the editor and reviewers viewed the work timely and of general interest to the broad scientific audience of *GigaScience*. We found the comments helpful and interesting and have carefully read through and acted on all reviewer suggestions (all changes highlighted in the newly submitted version), and explain our responses, point-by-point, in this letter (text in blue preceded by ' >> ').

Many thanks for your time,

We look forward to hearing from you.

Yours sincerely,

Paula Arribas & Brent C. Emerson (on behalf of co-authors)

Editor comments:

Dear Dr Arribas,

Your manuscript "*Toward global integration of biodiversity big data: a harmonised metabarcode data generation module for terrestrial arthropods*" (Review Article, GIGA-D-21-00420) has been assessed by our reviewers. Based on these reports, and my own assessment as Editor, I am pleased to inform you that it is potentially acceptable for publication in GigaScience, once you have carried out some essential revisions suggested by our reviewers. Their reports are below. Please note, reviewer #2 requested a document with line numbers during review, and I added those to your word document and re-uploaded it to Editorial Manager - please download the version with line numbers from EM to see which lines the reviewer refers to.

>> Thank you very much for the overall positive evaluation of our manuscript. We have revised the text to incorporate the suggestions of the reviewers. We are very grateful for the time that they have dedicated to reviewing our work, and their suggestions have improved clarity and provide for a more polished manuscript. Please see below for details.

Reviewer reports:

Reviewer #1: Comments to Authors (please, see pdf for a better format).

I found the manuscript "Toward global integration of biodiversity big data: a harmonised metabarcode data generation module for terrestrial arthropods" very interesting and useful.

I think that a review of the current metabarcoding methods and techniques is timely and the provided structure in modules seems to work well, adding a lot of value to the work. The suggested module framework can be very valuable, especially considering it leaves options for customization.

I personally think that the literature review is quite complete, and the information reported give the reader a good picture of the topic.

>> Thank you for your positive evaluation, and for the time you have dedicated improving the clarity of our manuscript, we appreciate it.

I have a few suggestions that I hope may help making the paper even clearer and more useful to the reader. For example, I think the structure of the paper should match the structure of the figure, with a clear subdivision in modules and submodules (i.e., chapters and subchapters). While it surely is not the ideal behaviour, we are all aware that many readers will probably skim through the article to the different modules they are interested on. A more defined chapter structure of the article will make it more useful to a wider audience.

>> We think that the limited way we integrated between the figure and text may have created some confusion, and thus we have clarified this aspect in the new version. Please see below the details in the specific comment.

The writing is clear, and the article is well-written. I hope the authors will forgive me if I spent a bit of time making (probably picky) changes to the wording, especially in the conclusion. This is only because I think the paper really has value and the conclusion will be one of the most-read parts of the article once published.

>> We don't think this is picky at all, and appreciate the time you have invested in our work. All the suggestions have been incorporated into the new version.

I have a few main comments and some minor changes (below), but I think the article should be accepted after their corrections.

Please, add continuous line numbers when resubmitting.

>> Done.

Main comments:

Module 1: Sample acquisition.

- At page 7, the authors start with the "Sample acquisition" chapter, which is their "Module 1" in the figure. The title of this chapter should be "Module 1: Sample acquisition". Here the reader, after a first introductory paragraph where a sample is defined (see next comment), should be able to find three subsections: submodule 1.1: malaise traps, submodule 1.2: pitfall traps. This proposed structure could also help readers focus on the part they are interested in. For example, if a researcher is using only pitfall traps, they will go directly to submodule 1.2. What about submodule 1.3? It appears in the figure but not in the text. If the figure is just an example of submodules that can be added, the authors should state that.

>> Thank you for pointing out this potential source of confusion. Please note that the section “Sample acquisition” does not correspond with Module 1 in Figure 1. We have clarified in the text and figure legend that Figure 1 is a schematic representation of the modular structure proposed for building a harmonised framework for the generation of metabarcode data for different fractions of terrestrial animals (modified text, lines 136). Our manuscript proposes this framework, and develops one such module: the terrestrial arthropods module, which focused on harmonisation for this biodiversity fraction.

The rationale for the structure of the terrestrial arthropod module text is that, once the justification of the general need for this module is developed in the text, we (i) review the existing literature pertaining to the five main steps (as subsections) and based on that, (ii) propose one or several submodules per step, providing a summary table for each submodule. We have reworded the text and headings (e.g. modified text, lines 136, 149, 186) to clarify this. We agree with the reviewer that many readers will probably skim through the article to the different submodules they are interested in, so thus we provide a summary table for each proposed submodule. So, for the first step (1. Sample acquisition step) of the terrestrial arthropod module we propose the 1.1 Malaise trapping sample

acquisition submodule (Table 1) and the 1.2 Pitfall trapping sample acquisition submodule (Table 2). Additional submodules can be further developed within this module (e.g. 1.3, 1.4…).

- This module start saying that it's important to have a "sample definition", and I totally agree. However, the authors do not provide one. It is true that a sample definition is strongly linked to the collection technique, but I think that we still require a sample definition, and I think the authors should be able to provide one. In my opinion, all the information necessary for a definition is in the text, it just need to be summarised. For example, a sample is composed by the arthropods, but also by their preservation, and the associated metadata. If any of these factors is missing, the sample is not fit for metabarcoding. It would be useful to know what else is really required for a sample to be defined as such. This sample definition should be after the first sentence and prior to the second one.

>> Please see the previous comment, and note that information regarding the sample definition for each submodule is summarized in the corresponding table for each (i.e. Table 1 for the 1.1 Malaise trapping sample acquisition submodule and Table 2 for the 1.2 Pitfall trapping sample acquisition submodule).

- After the first introductory aspects on definition of a sample, the authors cite the work of Montgomery and colleagues, where seven different collection methods are listed. The authors state that these methods provide "an appropriate platform from which to develop sample acquisition submodules". After reading this, I would have expected the authors to provide a submodule on EACH of these seven methods. Instead, only malaise traps and pitfall traps are presented. By doing this, the authors are either contradicting themselves and the work of Montgomery, or they are not clear on the reason they decide to report only two methods. Are the authors suggesting that, of the seven sampling techniques proposed, only malaise traps and pitfall traps are good for metabarcoding? Or are you suggesting that these two alone can provide good-enough results? Or again that, while all seven techniques are good, the authors are explaining only two? (If so, why?). In any case, this should be explained in detail.

>> We do not suggest that only malaise traps and pitfall traps are good for metabarcoding, rather we suggest that these two provide a useful minimum set for providing broad representation. We are perhaps not clear enough on this in our original text, and we have

now sought to be clearer on this point (new text, lines 261). We discuss that the seven arthropod sampling methods proposed by the review of Montgomery et al. 2021 are a solid basis to develop submodules within the Sample Acquisition step of the terrestrial arthropod module. We then review existing arthropod metabarcoding literature, and identify malaise traps as the most relevant in terms of its (i) dominant use compared to the other sampling methods, and (i) lack of harmonisation. We then identify pitfall trapping as complementary to malaise trapping because it is directed to less dispersive ground active species (modified text, lines 260). Please note that we also further encourage the development of additional submodules within the terrestrial arthropod module (new text, lines 566). However, we consider malaise and pitfall trapping to be an appropriate minimum set.

- In addition to this, I would separate the sampling techniques from the metadata collection, or it could get very repetitive. In fact, independently of the collection method, the metadata information should always accompany the arthropods sample. For example, why in table 1.2 is not reported "Extreme weather events during trapping"? This is very important for pitfall traps, too. Indeed, a major rainfall could dilute the preservative or even make the trap overflow (with relative risk of losing specimens). While a very hot weather is known to cause evaporation, with the risk of drying the trap. The authors mention this in the text, but not in the table.

>> We agree with the reviewer that Sampling Event metadata can be repetitive across sample acquisition submodules. However, we feel strongly that it is so should be considered as an essential part of sample acquisition, and so we prefer to maintain it in each sample acquisition submodule. We agree with the reviewer the ‘Extreme weather events during trapping’ metadata is a key point also for submodule 1.2 and have incorporated the info in the corresponding Table 2.

- In the same table, since it is reported the solution % for ethanol, also the glycol solution % should be reported. When using glycol in pitfall traps, the percentage should be lower than 95% (ideally between 40%/80% due to the viscosity of this preservative). At a 95% concentration, glycol may be so viscous that insect are not entirely submerged when they fall in the trap.

>> We have clarified the concentration of the propylene glycol in Table 2.

Module 2: Sample processing.

- As for the previous module, submodule paragraphs would be very helpful.

>> Please see our previous comment on this.

- I think the authors make an interesting point on the fact that size-sorting is not as necessary as one would think when deeper sequencing depth is an option. However, I have some issues with the explanations for this statement. The authors state that "increasing sequencing depth by 3-4 fold" to a "sufficient sequencing depth", together with "reasonable size ranges" make size-sorting superfluous. All these terms, unfortunately, are extremely subjective and do not enable the reader to understand when a sufficient sequencing depth is reached. Telling the reader that they need a "sufficient sequencing depth" to be able to ignore size-sorting is a tautology: it is obvious that if the sequencing depth is sufficient your work is good. In my opinion, the question readers would ask themselves is: what is a good sequencing depth in order for me to avoid size-sorting as the authors suggest? It is mentioned an increase of 3-4 fold, but that is relative to the whatever number of reads you had to start with. It would probably be useful for the reader to understand what platform the authors are referring to at this stage, but that would also require the authors to explain how many samples they would process per run. Depending on the work conducted, an increase of 3-4 folds in sequencing depth may mean the operator has to move from a MiSeq to a NovaSeq, for example. Or reduce the number of samples processed on each run (or their replicates). These factors should be considered, or at least mentioned, when suggesting that a higher sequencing depth is better than size-sorting. If the reader makes it to the end of the modules, they will notice this topic is mentioned at page 21. I think, however, that the correlation between sample processing and sequencing depth is extremely important and should be explained in this module.

>> The reviewer makes a good point, and we think the simplest way to deal with it is to remove the explicit mention of 3-4 fold. Indeed, 3-4 fold is specifically relevant to the reference being cited. We agree with the reviewer that the increase in sequencing depth will depend on project specific parameters, and thus there is no magic number. We thus make the general point that increased sequencing depth is an alternative to size sorting (modified text, lines 311). We also have reworded the text to direct the reader to the discussion of step 4 (Amplification, library preparation and sequencing step section) on the sequencing depth (new text, lines 315).

I agree that size-sorting is terribly time-consuming and therefore expensive; however, having to run your samples on two runs instead of one to get a better sequencing depth would be probably more expensive. I am not sure if it can be useful to the authors, but Piper and colleagues (GigaScience, 8, 2019, 1-22, doi: 10.1093/gigascience/giz092, which is cited as reference number 7) provide a table with the costs and Gb output for each platform. This may be useful to give a reader an idea of what a good sequencing depth can be. Or link the readers to the page 21 explanation of the average reads-per-specimen expected in each sample. Otherwise, a possibly simpler solution could be to provide the reader with a method to determine what a good sequencing depth looks like. For example, a taxa recovery graph that reaches plateau has been considered a valid and easy test to determine this (Hajibabaei et al. 2019 - PLoS One. 2019; 14(9): e0220953. doi: 10.1371/journal.pone.0220953).

>> Thank you for these relevant references. We have included them in the corresponding section (new text, lines 505).

Minor changes:

Page 5, line 4: remove "are". It should read: "by placing different fractions of terrestrial diversity at the core of each "module".

>> Done.

Page 6, first 8 lines of the "Harmonisation for the metabarcoding of terrestrial arthropods" paragraph: Compared to the rest of the introduction, this paragraph could be improved both in form and in content. It seems a few different topics have just been put together, with an isolated sentence for each, without going in depth enough and without linking the sentences to each other. I suggest the authors either rewrite this paragraph or simply list the reasons why arthropods assessment is useful (e.g., biodiversity assessment, conservation of declining species, monitoring of invasives). As per the form, the use of terms such as "overwhelming" and "tremendous" could be avoided (a bit too subjective), as it should be the repetition of the word "present" at line 2.

>> We have reworded this paragraph according to reviewer suggestions (modified text, lines 151).

Page 6, last line: remove "in". It should read: "comparable to standard methods of arthropod monitoring".

>> Done.

Page 7, line 5: Close parenthesis after the references and remove the comma.

>> Done.

Page 7, first line of "Sample acquisition": "Starting point" instead of "departure point".

>> Done.

Page 15, "DNA extraction" Chapter, line 10: The authors mention the "taxonomic content of samples" and in bracket give the definition of OTUs. This can be confusing for the reader. The taxonomic content of a sample is not necessarily defined by OTUs, but could be extrapolated using ASVs (amplicon sequence variants). Since the authors are referring to a specific paper they are referencing, I suggest to change the sentence to: "When assessing the recovered taxonomic content of samples using operational taxonomic units (OTUs), intact samples performed at least comparable.

>> Changed.

Page 16: The authors suggest that 100-200 µl of DNA extraction buffer can be considered appropriate for harmonisation. This gives the impression the authors are suggesting to use only 200 units of buffer when performing the DNA extraction. In my experience, an average pitfall trap that has been in the field for a week an contains even just 2 bees and 2 beetles (very unlikely) can easily require almost 1 ml of buffer when using a non-destructive DNA extraction method. As the authors stated a few sentences earlier, this is a large volume of buffer. Then why suggesting that 100-200 µl is enough? Was this referring to the use of just 100-200 µl as a subsample to purify from the overall volume used? If so, the sentence should read something like:

"Given this consideration, typical commercial kit extraction volumes of 100-200 µl can be considered an appropriate sub-sampling volume for subsequent purification."

>> Yes, that was our point. Changed (modified text, lines 387).

Page 17, Chapter 4: gene names should be italicised. Correct to: "Cytochrome c oxidase subunit I barcode region". Please, note that "subunit I" is not part of the name and should not be italicised.

>> Done.

Page 18, Line 3: I would break the sentence in two: "The BF3 fragment (418 bp) provides better taxonomic resolution than other overlapping fragments. Furthermore, primers within this region are also unaffected by slippage, and provide maximum overlap across already published studies."

>> Done.

Page 18, Line 18: Reference is missing, check "ref".

>> Included.

Page 18, Line 20: My understanding is that the proofreading activity of a polymerase is the 3′→5′ exonuclease activity. I am not sure what the "non" refers to. I think it should read: "their proofreading activity (3′→5′ exonuclease activity)"

>> Corrected.

Page 23, "Conclusion" Line 1: No need to give both the full name and the abbreviation for wocDNA, since this was done previously. The authors can pick one.

>> Done.

Page 23, "Conclusion" Line 7: "address this issue".

>> Done.

Page 23, "Conclusion" Line 8 and 11: the use of the term "canalization", while technically correct, seems a bit odd and adds unnecessary jargon, especially considering the conclusion will be read by most readers. I would suggest changing this term.

>> Changed.

Page 23, "Conclusion" Line 13 and 14: "submodule", "modular" and "modules" in the same sentence makes it very hard to read.. A possible solution could be:

"the flexible structure we presented here seeks to broaden the applicability of a modular framework within the wocDNA metabarcoding community."

>> Replaced.

Page 23, "Conclusion" Line 18: Again, it is a bit repetitive to mention the submodule structure of the module. If it is a submodule, then it is already given that is part of the module. I would rephrase by removing "module".

>> Removed.

Reviewer #2: The manuscript makes a well-argued case for the adoption of consistent metabarcoding data generation workflows (harmonisation) for inventorying macro-biodiversity, within a modular framework, to enable larger-scale analyses that incorporate multiple datasets - and this is clearly a good idea. To do this, the authors review the relevant literature, and based on this, provide sets of workflow recommendations, at five key data generation steps, within a proposed terrestrial arthropod metabarcoding module.

The paper is largely well written and easy to follow (apart from some parts detailed in the line-by-line comments below). The authors have done an excellent job of reviewing the relevant literature, and the manuscript is packed with useful workflow recommendations for metabarcoding of terrestrial invertebrates. A particularly helpful aspect is the consideration of all data generation steps, from initial sampling through to the storage of sequence data and metadata.

One possible omission is that almost no mention is made of arthropods living below ground, which is an important component of terrestrial arthropod biodiversity, with another set of sampling methods and considerations. Given that the manuscript focuses on workflows for "terrestrial arthropods", I think it should at least be mentioned that that sampling for soil arthropod metabarcoding would be another submodule, but is not considered in this manuscript. Similarly, it might be helpful to suggest other modules that could or should be developed, within the conclusion?

>> Thank you for your assessment, and for the general point you raise in your last paragraph. We fully agree on the importance of considering soil arthropods. In this manuscript, we reviewed the literature and focused on developing two submodules that we find to have more immediate relevance, in terms of their already popular implementation (i.e. malaise traps), complementarity (i.e. pitfall trapping) and lack of harmonisation. Soil arthropods are an obvious candidate for further submodule development. We agree that it is worth suggesting different submodules within the conclusions that could or should be developed within the terrestrial arthropod module, and we explicitly mention soil arthropods as an important candidate group (new text, lines 566).

Are these modules going to exist anywhere apart from within this manuscript and subsequent manuscripts? It might be helpful to have a website that collects all these modules into one place for easy access, somewhat like the Earth microbiome project website.

>> We plan to place submodules in the iBioGen project webpage (https://www.ibiogen.eu/deliverables.html), together with this and subsequent manuscripts on this topic. Additionally, we have prepared a video explaining the details of the submodules proposed in this manuscript. This video is already available via the iBioGen webpage (see https://www.ibiogen.eu/dissemination.html). Please note, it still requires final editions to accommodate modifications resulting from this review process. Once updates have been implemented and our manuscript accepted, it will be disseminated through the social media of the iBioGen project, and the authors.

L 34: For inventorying biodiversity? For compiling biodiversity inventories?

>> Changed.

L 79: It is unclear whether "metabarcode inventory data" means the data resulting from metabarcoding analyses, or the data about metabarcoding methods/workflows?

>> Clarified.

L 89: I think "global microbial initiatives" is missing something. Global microbial diversity assessment initiatives? Also, I'm not sure "(even if data generation has been centralised)" is needed.

>> Reworded.

L 94: What are eDNA initiatives, as opposed to metabarcoding initiatives?

>> Clarified.

L 98: "one of the most heterogeneous groups in terms of body size"?

>> Done.

L 99: I think it would be clearer to use "inventorying of" (i.e. compiling an inventory), rather than "inventory". (Inventorying is used elsewhere, e.g. L 108, 166).

>> Reworded.

L 110: "calibration and so" seems unnecessary.

>> Removed.

L 111: It's unclear to me why catalysis of a GO network is the key challenge. Perhaps consistent workflows are implicit in a GO network? But consistent workflows could exist without a GO network too. Can you clarify how a GO network helps?

>> We agree with the reviewer and have reworded to clarify (modified text, lines 112).

L 119-122: Arguably, bioinformatic processing of raw sequence data into processed data is another key step (depending on whether "data" is the raw sequence data, or processed OTU/ASV data). Evidently, this is not within the scope of the manuscript, but it might be worth mentioning somewhere that post-sequencing aspects of metabarcoding workflows can also vary a lot, resulting in incomparable datasets. However, this is less problematic because one can theoretically re-process the sequence data from different studies in a consistent manner.

>> We fully agree with the reviewer on the importance of harmonisation for the bioinformatic processing of raw sequence data, and we have recently published specifically on this topic (Creedy et al. 2022 (our reference 34). We have now mentioned this aspect in the manuscript, as suggested by the reviewer (new text, lines 87).

L 140-142: This sentence is very confusing. "long-view" should probably be "long-term goal"; "synthetic analyses" sounds like analyses of synthetic (artificial or man-made) data; and I'm not sure what "a function of any collateral costs" means. Please rephrase.

>> Reworded.

L 144: minimal compromise, if any?

>> Reworded.

L 150: The declines of insects (plural) are now a very real and serious threat?

>> Corrected.

L 161: inventorying arthropod biodiversity?

>> Reworded.

L 162: Remove "in".

>> Done.

L 183-184: panacea? Might be better to say "no one method detecting the entire arthropod diversity within a site"

>> Done.

L 273: I'm not sure "for harmonisation" is needed here.

>> Removed.

L 321: Photographing of invertebrate samples is an excellent idea!

>> Thanks.

L 330: Would there be a benefit to trying to orient all the specimens in the same way, for potential future visual-based identifications? (probably time-consuming though).

>> This is ideal but very time-consuming in most types of arthropod bulk samples, that is why we did not include it.

L 307: "4mm sieve pooled 1:10 to 2:10" is unclear. Does it mean, the < 4mm and > 4mm fractions are pooled together at a ratio of 1:10 to 2:10? Which fraction is the higher ratio? Please clarify.

>> Clarified.

L 337: What is a SuperGO?

>> Within the spatially led terrestrial GO network that we propose in Arribas et al. 2021, SuperGOs are sites where molecular community data is more intensively generated at both the temporal and the genomic axes, consistent with the idea of “model ecosystems” (Davies et al., 2012, 2014). This has now been clarified in the text (new text, lines 351).

L 398-405: "COI-bcr" is unnecessary, only used in this paragraph. "COI barcode" is used on line 408 to mean the same thing, and is clearer. I suggest replacing "COI-bcr" on lines 401 and 405 with "COI barcode" and COI barcode region", respectively.

>> Done.

L 405-406: This sentence should be rephrased. Multiple COI-targeted primer sets … demonstrated to efficiently characterise arthropods … particularly those with certain degenerate positions?

>> Reworded.

L 407: see Figure 2 in Elbrecht et al.? Should "second half" be 3' (prime)?

>> Done.

L 408-412: Can you provide citation for claims about BF3, and for primers BF2, III_B_F, Fol-degen-rev? I think the "primers within this region…" statement should be qualified with a word such as "published" or "tested". Maximum overlap of what among already published studies? (COI regions?) Do these primers have any limitations in terms of taxonomic coverage?

>> We have now provided references, and specifically cite Figure 2 in Elbrecht et al., [37] for a summary of the sequence, original citation and efficiency of each primer set (modified text, lines 424).

L 412: Why "eDNA metabarcoding" here, but just "metabarcoding" everywhere else?

>> Removed.

L 424: Citation missing?

>> Included.

L 471: Why would that be so? (Lower cost?)

>> Yes, clarified.

Table 5: What is BC3 fragment? I'm not sure how useful it is to recommend "degenerate primers" here - presumably it is certain specific COI-targeted degenerate primers that are recommended, not degenerate primers in general, in which case should they be listed here?

>> We have added additional details to Table 5 to clarify these aspects already included in the text.

L 533: I'm not sure what canalisation means. Replace with harmonisation?

>> Replaced.

L 539-540: "of modules" seems repetitive (of submodules) and unnecessary.

>> Removed.
