# Supplementary material for: Toward global integration of biodiversity big data: a harmonized metabarcode data generation module for terrestrial arthropods
Source: Gigascience. 2022 Jul 19;11:giac065. doi: 10.1093/gigascience/giac065 (PMC9295367; doi:10.1093/gigascience/giac065)
Supplement: giac065_Response_to_Reviewer_Comments_Original_Submission [file giac065_response_to_reviewer_comments_original_submission.pdf]

Author's Response To Reviewer Comments

Close

Dear Editor,
Many thanks for your comments with respect to our manuscript entitled “Toward global integration of biodiversity big data: a harmonised metabarcode data generation module for terrestrial arthropods”. We are pleased that the editor and reviewers viewed the work timely and of general interest to the broad scientific audience of GigaScience. We found the comments helpful and interesting and have carefully read through and acted on all reviewer suggestions (all changes highlighted in the newly submitted version), and explain our responses, point-by-point, in this letter (text in blue preceded by ' >> ').
Many thanks for your time,
We look forward to hearing from you.
Yours sincerely,
Paula Arribas & Brent C. Emerson (on behalf of co-authors)

Editor comments:

Dear Dr Arribas,
Your manuscript "Toward global integration of biodiversity big data: a harmonised metabarcode data generation module for terrestrial arthropods" (Review Article, GIGA-D-21-00420) has been assessed by our reviewers. Based on these reports, and my own assessment as Editor, I am pleased to inform you that it is potentially acceptable for publication in GigaScience, once you have carried out some essential revisions suggested by our reviewers. Their reports are below. Please note, reviewer #2 requested a document with line numbers during review, and I added those to your word document and re-uploaded it to Editorial Manager - please download the version with line numbers from EM to see which lines the reviewer refers to.
>> Thank you very much for the overall positive evaluation of our manuscript. We have revised the text to incorporate the suggestions of the reviewers. We are very grateful for the time that they have dedicated to reviewing our work, and their suggestions have improved clarity and provide for a more polished manuscript. Please see below for details.

Reviewer reports:
Reviewer #1: Comments to Authors (please, see pdf for a better format).

I found the manuscript "Toward global integration of biodiversity big data: a harmonised metabarcode data generation module for terrestrial arthropods" very interesting and useful.
I think that a review of the current metabarcoding methods and techniques is timely and the provided structure in modules seems to work well, adding a lot of value to the work. The suggested module framework can be very valuable, especially considering it leaves options for customization.
I personally think that the literature review is quite complete, and the information reported give the reader a good picture of the topic.
>> Thank you for your positive evaluation, and for the time you have dedicated improving the clarity of our manuscript, we appreciate it.
I have a few suggestions that I hope may help making the paper even clearer and more useful to the reader. For example, I think the structure of the paper should match the structure of the figure, with a clear subdivision in modules and submodules (i.e., chapters and subchapters). While it surely is not the ideal behaviour, we are all aware that many readers will probably skim through the article to the different modules they are interested on. A more defined chapter structure of the article will make it more useful to a wider audience.
>> We think that the limited way we integrated between the figure and text may have created some confusion, and thus we have clarified this aspect in the new version. Please see below the details in the specific comment.
The writing is clear, and the article is well-written. I hope the authors will forgive me if I spent a bit of

time making (probably picky) changes to the wording, especially in the conclusion. This is only because I think the paper really has value and the conclusion will be one of the most-read parts of the article once published.
>> We don’t think this is picky at all, and appreciate the time you have invested in our work. All the suggestions have been incorporated into the new version.
I have a few main comments and some minor changes (below), but I think the article should be accepted after their corrections.
Please, add continuous line numbers when resubmitting.
>> Done.
Main comments:
Module 1: Sample acquisition.
- At page 7, the authors start with the "Sample acquisition" chapter, which is their "Module 1" in the figure. The title of this chapter should be "Module 1: Sample acquisition". Here the reader, after a first introductory paragraph where a sample is defined (see next comment), should be able to find three subsections: submodule 1.1: malaise traps, submodule 1.2: pitfall traps. This proposed structure could also help readers focus on the part they are interested in. For example, if a researcher is using only pitfall traps, they will go directly to submodule 1.2. What about submodule 1.3? It appears in the figure but not in the text. If the figure is just an example of submodules that can be added, the authors should state that.
>> Thank you for pointing out this potential source of confusion. Please note that the section “Sample acquisition” does not correspond with Module 1 in Figure 1. We have clarified in the text and figure legend that Figure 1 is a schematic representation of the modular structure proposed for building a harmonised framework for the generation of metabarcode data for different fractions of terrestrial animals (modified text, lines 136). Our manuscript proposes this framework, and develops one such module: the terrestrial arthropods module, which focused on harmonisation for this biodiversity fraction. The rationale for the structure of the terrestrial arthropod module text is that, once the justification of the general need for this module is developed in the text, we (i) review the existing literature pertaining to the five main steps (as subsections) and based on that, (ii) propose one or several submodules per step, providing a summary table for each submodule. We have reworded the text and headings (e.g. modified text, lines 136, 149, 186) to clarify this. We agree with the reviewer that many readers will probably skim through the article to the different submodules they are interested in, so thus we provide a summary table for each proposed submodule. So, for the first step (1. Sample acquisition step) of the terrestrial arthropod module we propose the 1.1 Malaise trapping sample acquisition submodule (Table 1) and the 1.2 Pitfall trapping sample acquisition submodule (Table 2). Additional submodules can be further developed within this module (e.g. 1.3, 1.4…).
- This module start saying that it's important to have a "sample definition", and I totally agree. However, the authors do not provide one. It is true that a sample definition is strongly linked to the collection technique, but I think that we still require a sample definition, and I think the authors should be able to provide one. In my opinion, all the information necessary for a definition is in the text, it just need to be summarised. For example, a sample is composed by the arthropods, but also by their preservation, and the associated metadata. If any of these factors is missing, the sample is not fit for metabarcoding. It would be useful to know what else is really required for a sample to be defined as such. This sample definition should be after the first sentence and prior to the second one.
>> Please see the previous comment, and note that information regarding the sample definition for each submodule is summarized in the corresponding table for each (i.e. Table 1 for the 1.1 Malaise trapping sample acquisition submodule and Table 2 for the 1.2 Pitfall trapping sample acquisition submodule).
- After the first introductory aspects on definition of a sample, the authors cite the work of Montgomery and colleagues, where seven different collection methods are listed. The authors state that these methods provide "an appropriate platform from which to develop sample acquisition submodules". After reading this, I would have expected the authors to provide a submodule on EACH of these seven methods. Instead, only malaise traps and pitfall traps are presented. By doing this, the authors are either contradicting themselves and the work of Montgomery, or they are not clear on the reason they decide to report only two methods. Are the authors suggesting that, of the seven sampling techniques proposed, only malaise traps and pitfall traps are good for metabarcoding? Or are you suggesting that these two alone can provide good-enough results? Or again that, while all seven techniques are good, the authors are explaining only two? (If so, why?). In any case, this should be explained in detail.
>> We do not suggest that only malaise traps and pitfall traps are good for metabarcoding, rather we suggest that these two provide a useful minimum set for providing broad representation. We are perhaps not clear enough on this in our original text, and we have now sought to be clearer on this point (new text, lines 261). We discuss that the seven arthropod sampling methods proposed by the review of Montgomery et al. 2021 are a solid basis to develop submodules within the Sample Acquisition step of the terrestrial arthropod module. We then review existing arthropod metabarcoding literature, and

identify malaise traps as the most relevant in terms of its (i) dominant use compared to the other sampling methods, and (i) lack of harmonisation. We then identify pitfall trapping as complementary to malaise trapping because it is directed to less dispersive ground active species (modified text, lines 260). Please note that we also further encourage the development of additional submodules within the terrestrial arthropod module (new text, lines 566). However, we consider malaise and pitfall trapping to be an appropriate minimum set.
- In addition to this, I would separate the sampling techniques from the metadata collection, or it could get very repetitive. In fact, independently of the collection method, the metadata information should always accompany the arthropods sample. For example, why in table 1.2 is not reported "Extreme weather events during trapping"? This is very important for pitfall traps, too. Indeed, a major rainfall could dilute the preservative or even make the trap overflow (with relative risk of losing specimens). While a very hot weather is known to cause evaporation, with the risk of drying the trap. The authors mention this in the text, but not in the table.
>> We agree with the reviewer that Sampling Event metadata can be repetitive across sample acquisition submodules. However, we feel strongly that it is so should be considered as an essential part of sample acquisition, and so we prefer to maintain it in each sample acquisition submodule. We agree with the reviewer the 'Extreme weather events during trapping' metadata is a key point also for submodule 1.2 and have incorporated the info in the corresponding Table 2.
- In the same table, since it is reported the solution % for ethanol, also the glycol solution % should be reported. When using glycol in pitfall traps, the percentage should be lower than 95% (ideally between 40%/80% due to the viscosity of this preservative). At a 95% concentration, glycol may be so viscous that insect are not entirely submerged when they fall in the trap.
>> We have clarified the concentration of the propylene glycol in Table 2.
Module 2: Sample processing.
- As for the previous module, submodule paragraphs would be very helpful.
>> Please see our previous comment on this.
- I think the authors make an interesting point on the fact that size-sorting is not as necessary as one would think when deeper sequencing depth is an option. However, I have some issues with the explanations for this statement. The authors state that "increasing sequencing depth by 3-4 fold" to a "sufficient sequencing depth", together with "reasonable size ranges" make size-sorting superfluous. All these terms, unfortunately, are extremely subjective and do not enable the reader to understand when a sufficient sequencing depth is reached. Telling the reader that they need a "sufficient sequencing depth" to be able to ignore size-sorting is a tautology: it is obvious that if the sequencing depth is sufficient your work is good. In my opinion, the question readers would ask themselves is: what is a good sequencing depth in order for me to avoid size-sorting as the authors suggest? It is mentioned an increase of 3-4 fold, but that is relative to the whatever number of reads you had to start with. It would probably be useful for the reader to understand what platform the authors are referring to at this stage, but that would also require the authors to explain how many samples they would process per run. Depending on the work conducted, an increase of 3-4 folds in sequencing depth may mean the operator has to move from a MiSeq to a NovaSeq, for example. Or reduce the number of samples processed on each run (or their replicates). These factors should be considered, or at least mentioned, when suggesting that a higher sequencing depth is better than size-sorting. If the reader makes it to the end of the modules, they will notice this topic is mentioned at page 21. I think, however, that the correlation between sample processing and sequencing depth is extremely important and should be explained in this module.
>> The reviewer makes a good point, and we think the simplest way to deal with it is to remove the explicit mention of 3-4 fold. Indeed, 3-4 fold is specifically relevant to the reference being cited. We agree with the reviewer that the increase in sequencing depth will depend on project specific parameters, and thus there is no magic number. We thus make the general point that increased sequencing depth is an alternative to size sorting (modified text, lines 311). We also have reworded the text to direct the reader to the discussion of step 4 (Amplification, library preparation and sequencing step section) on the sequencing depth (new text, lines 315).
I agree that size-sorting is terribly time-consuming and therefore expensive; however, having to run your samples on two runs instead of one to get a better sequencing depth would be probably more expensive. I am not sure if it can be useful to the authors, but Piper and colleagues (GigaScience, 8, 2019, 1-22, doi: 10.1093/gigascience/giz092, which is cited as reference number 7) provide a table with the costs and Gb output for each platform. This may be useful to give a reader an idea of what a good sequencing depth can be. Or link the readers to the page 21 explanation of the average reads-per-specimen expected in each sample. Otherwise, a possibly simpler solution could be to provide the reader with a method to determine what a good sequencing depth looks like. For example, a taxa recovery graph that reaches plateau has been considered a valid and easy test to determine this (Hajibabaei et al. 2019 - PLoS One. 2019; 14(9): e0220953. doi: 10.1371/journal.pone.0220953).

>> Thank you for these relevant references. We have included them in the corresponding section (new text, lines 505).

Minor changes:

Page 5, line 4: remove "are". It should read: "by placing different fractions of terrestrial diversity at the core of each "module".

>> Done.

Page 6, first 8 lines of the "Harmonisation for the metabarcoding of terrestrial arthropods" paragraph: Compared to the rest of the introduction, this paragraph could be improved both in form and in content. It seems a few different topics have just been put together, with an isolated sentence for each, without going in depth enough and without linking the sentences to each other. I suggest the authors either rewrite this paragraph or simply list the reasons why arthropods assessment is useful (e.g., biodiversity assessment, conservation of declining species, monitoring of invasives). As per the form, the use of terms such as "overwhelming" and "tremendous" could be avoided (a bit too subjective), as it should be the repetition of the word "present" at line 2.

>> We have reworded this paragraph according to reviewer suggestions (modified text, lines 151).

Page 6, last line: remove "in". It should read: "comparable to standard methods of arthropod monitoring".

>> Done.

Page 7, line 5: Close parenthesis after the references and remove the comma.

>> Done.

Page 7, first line of "Sample acquisition": "Starting point" instead of "departure point".

>> Done.

Page 15, "DNA extraction" Chapter, line 10: The authors mention the "taxonomic content of samples" and in bracket give the definition of OTUs. This can be confusing for the reader. The taxonomic content of a sample is not necessarily defined by OTUs, but could be extrapolated using ASVs (amplicon sequence variants). Since the authors are referring to a specific paper they are referencing, I suggest to change the sentence to: "When assessing the recovered taxonomic content of samples using operational taxonomic units (OTUs), intact samples performed at least comparable.

>> Changed.

Page 16: The authors suggest that 100-200 µl of DNA extraction buffer can be considered appropriate for harmonisation. This gives the impression the authors are suggesting to use only 200 units of buffer when performing the DNA extraction. In my experience, an average pitfall trap that has been in the field for a week an contains even just 2 bees and 2 beetles (very unlikely) can easily require almost 1 ml of buffer when using a non-destructive DNA extraction method. As the authors stated a few sentences earlier, this is a large volume of buffer. Then why suggesting that 100-200 µl is enough? Was this referring to the use of just 100-200 µl as a subsample to purify from the overall volume used? If so, the sentence should read something like:

"Given this consideration, typical commercial kit extraction volumes of 100-200 µl can be considered an appropriate sub-sampling volume for subsequent purification."

>> Yes, that was our point. Changed (modified text, lines 387).

Page 17, Chapter 4: gene names should be italicised. Correct to: "Cytochrome c oxidase subunit I barcode region". Please, note that "subunit I" is not part of the name and should not be italicised.

>> Done.

Page 18, Line 3: I would break the sentence in two: "The BF3 fragment (418 bp) provides better taxonomic resolution than other overlapping fragments. Furthermore, primers within this region are also unaffected by slippage, and provide maximum overlap across already published studies."

>> Done.

Page 18, Line 18: Reference is missing, check "ref".

>> Included.

Page 18, Line 20: My understanding is that the proofreading activity of a polymerase is the 3′→5′ exonuclease activity. I am not sure what the "non" refers to. I think it should read: "their proofreading activity (3′→5′ exonuclease activity)"

>> Corrected.

Page 23, "Conclusion" Line 1: No need to give both the full name and the abbreviation for wocDNA, since this was done previously. The authors can pick one.

>> Done.

Page 23, "Conclusion" Line 7: "address this issue".

>> Done.

Page 23, "Conclusion" Line 8 and 11: the use of the term "canalization", while technically correct, seems a bit odd and adds unnecessary jargon, especially considering the conclusion will be read by most readers. I would suggest changing this term.

>> Changed.

Page 23, "Conclusion" Line 13 and 14: "submodule", "modular" and "modules" in the same sentence makes it very hard to read.. A possible solution could be:
"the flexible structure we presented here seeks to broaden the applicability of a modular framework within the wocDNA metabarcoding community."
>> Replaced.
Page 23, "Conclusion" Line 18: Again, it is a bit repetitive to mention the submodule structure of the module. If it is a submodule, then it is already given that is part of the module. I would rephrase by removing "module".
>> Removed.

Reviewer #2:

The manuscript makes a well-argued case for the adoption of consistent metabarcoding data generation workflows (harmonisation) for inventorying macro-biodiversity, within a modular framework, to enable larger-scale analyses that incorporate multiple datasets - and this is clearly a good idea. To do this, the authors review the relevant literature, and based on this, provide sets of workflow recommendations, at five key data generation steps, within a proposed terrestrial arthropod metabarcoding module.
The paper is largely well written and easy to follow (apart from some parts detailed in the line-by-line comments below). The authors have done an excellent job of reviewing the relevant literature, and the manuscript is packed with useful workflow recommendations for metabarcoding of terrestrial invertebrates. A particularly helpful aspect is the consideration of all data generation steps, from initial sampling through to the storage of sequence data and metadata.
One possible omission is that almost no mention is made of arthropods living below ground, which is an important component of terrestrial arthropod biodiversity, with another set of sampling methods and considerations. Given that the manuscript focuses on workflows for "terrestrial arthropods", I think it should at least be mentioned that that sampling for soil arthropod metabarcoding would be another submodule, but is not considered in this manuscript. Similarly, it might be helpful to suggest other modules that could or should be developed, within the conclusion?
>> Thank you for your assessment, and for the general point you raise in your last paragraph. We fully agree on the importance of considering soil arthropods. In this manuscript, we reviewed the literature and focused on developing two submodules that we find to have more immediate relevance, in terms of their already popular implementation (i.e. malaise traps), complementarity (i.e. pitfall trapping) and lack of harmonisation. Soil arthropods are an obvious candidate for further submodule development. We agree that it is worth suggesting different submodules within the conclusions that could or should be developed within the terrestrial arthropod module, and we explicitly mention soil arthropods as an important candidate group (new text, lines 566).
Are these modules going to exist anywhere apart from within this manuscript and subsequent manuscripts? It might be helpful to have a website that collects all these modules into one place for easy access, somewhat like the Earth microbiome project website.
>> We plan to place submodules in the iBioGen project webpage (https://www.ibiogen.eu/deliverables.html), together with this and subsequent manuscripts on this topic. Additionally, we have prepared a video explaining the details of the submodules proposed in this manuscript. This video is already available via the iBioGen webpage (see https://www.ibiogen.eu/dissemination.html). Please note, it still requires final editions to accommodate modifications resulting from this review process. Once updates have been implemented and our manuscript accepted, it will be disseminated through the social media of the iBioGen project, and the authors.
L 34: For inventorying biodiversity? For compiling biodiversity inventories?
>> Changed.
L 79: It is unclear whether "metabarcode inventory data" means the data resulting from metabarcoding analyses, or the data about metabarcoding methods/workflows?
>> Clarified.
L 89: I think "global microbial initiatives" is missing something. Global microbial diversity assessment initiatives? Also, I'm not sure "(even if data generation has been centralised)" is needed.
>> Reworded.
L 94: What are eDNA initiatives, as opposed to metabarcoding initiatives?
>> Clarified.
L 98: "one of the most heterogeneous groups in terms of body size"?
>> Done.
L 99: I think it would be clearer to use "inventorying of" (i.e. compiling an inventory), rather than "inventory". (Inventorying is used elsewhere, e.g. L 108, 166).

>> Reworded.
L 110: "calibration and so" seems unnecessary.
>> Removed.
L 111: It's unclear to me why catalysis of a GO network is the key challenge. Perhaps consistent workflows are implicit in a GO network? But consistent workflows could exist without a GO network too. Can you clarify how a GO network helps?
>> We agree with the reviewer and have reworded to clarify (modified text, lines 112).
L 119-122: Arguably, bioinformatic processing of raw sequence data into processed data is another key step (depending on whether "data" is the raw sequence data, or processed OTU/ASV data). Evidently, this is not within the scope of the manuscript, but it might be worth mentioning somewhere that post-sequencing aspects of metabarcoding workflows can also vary a lot, resulting in incomparable datasets. However, this is less problematic because one can theoretically re-process the sequence data from different studies in a consistent manner.
>> We fully agree with the reviewer on the importance of harmonisation for the bioinformatic processing of raw sequence data, and we have recently published specifically on this topic (Creedy et al. 2022 (our reference 34). We have now mentioned this aspect in the manuscript, as suggested by the reviewer (new text, lines 87).
L 140-142: This sentence is very confusing. "long-view" should probably be "long-term goal"; "synthetic analyses" sounds like analyses of synthetic (artificial or man-made) data; and I'm not sure what "a function of any collateral costs" means. Please rephrase.
>> Reworded.
L 144: minimal compromise, if any?
>> Reworded.
L 150: The declines of insects (plural) are now a very real and serious threat?
>> Corrected.
L 161: inventorying arthropod biodiversity?
>> Reworded.
L 162: Remove "in".
>> Done.
L 183-184: panacea? Might be better to say "no one method detecting the entire arthropod diversity within a site"
>> Done.
L 273: I'm not sure "for harmonisation" is needed here.
>> Removed.
L 321: Photographing of invertebrate samples is an excellent idea!
>> Thanks.
L 330: Would there be a benefit to trying to orient all the specimens in the same way, for potential future visual-based identifications? (probably time-consuming though).
>> This is ideal but very time-consuming in most types of arthropod bulk samples, that is why we did not include it.
L 307: "4mm sieve pooled 1:10 to 2:10" is unclear. Does it mean, the < 4mm and > 4mm fractions are pooled together at a ratio of 1:10 to 2:10? Which fraction is the higher ratio? Please clarify.
>> Clarified.
L 337: What is a SuperGO?
>> Within the spatially led terrestrial GO network that we propose in Arribas et al. 2021, SuperGOs are sites where molecular community data is more intensively generated at both the temporal and the genomic axes, consistent with the idea of "model ecosystems" (Davies et al., 2012, 2014). This has now been clarified in the text (new text, lines 351).
L 398-405: "COI-bcr" is unnecessary, only used in this paragraph. "COI barcode" is used on line 408 to mean the same thing, and is clearer. I suggest replacing "COI-bcr" on lines 401 and 405 with "COI barcode" and COI barcode region", respectively.
>> Done.
L 405-406: This sentence should be rephrased. Multiple COI-targeted primer sets … demonstrated to efficiently characterise arthropods … particularly those with certain degenerate positions?
>> Reworded.
L 407: see Figure 2 in Elbrecht et al.? Should "second half" be 3' (prime)?
>> Done.
L 408-412: Can you provide citation for claims about BF3, and for primers BF2, III_B_F, Fol-degen-rev? I think the "primers within this region…" statement should be qualified with a word such as "published" or "tested". Maximum overlap of what among already published studies? (COI regions?) Do these primers have any limitations in terms of taxonomic coverage?
>> We have now provided references, and specifically cite Figure 2 in Elbrecht et al., [37] for a

summary of the sequence, original citation and efficiency of each primer set (modified text, lines 424).
L 412: Why "eDNA metabarcoding" here, but just "metabarcoding" everywhere else?
>> Removed.
L 424: Citation missing?
>> Included.
L 471: Why would that be so? (Lower cost?)
>> Yes, clarified.
Table 5: What is BC3 fragment? I'm not sure how useful it is to recommend "degenerate primers" here - presumably it is certain specific COI-targeted degenerate primers that are recommended, not degenerate primers in general, in which case should they be listed here?
>> We have added additional details to Table 5 to clarify these aspects already included in the text.
L 533: I'm not sure what canalisation means. Replace with harmonisation?
>> Replaced.
L 539-540: "of modules" seems repetitive (of submodules) and unnecessary.
>> Removed.

Close
