# Supplementary material for: Toward global integration of biodiversity big data: a harmonized metabarcode data generation module for terrestrial arthropods
Source: Gigascience. 2022 Jul 19;11:giac065. doi: 10.1093/gigascience/giac065 (PMC9295367; doi:10.1093/gigascience/giac065)
Supplement: giac065_Reviewer_1_Report_Original_Submission — Francesco Martoni -- 1/23/2022 Reviewed [file giac065_reviewer_1_report_original_submission.pdf]

Reviewer Report

Title: Toward global integration of biodiversity big data: a harmonised metabarcode data generation module for terrestrial arthropods

Version: Original Submission **Date:** 1/23/2022

Reviewer name: Francesco Martoni

Reviewer Comments to Author:

Comments to Authors (please, see pdf for a better format).
I found the manuscript "Toward global integration of biodiversity big data: a harmonised metabarcode data generation module for terrestrial arthropods" very interesting and useful.
I think that a review of the current metabarcoding methods and techniques is timely and the provided structure in modules seems to work well, adding a lot of value to the work. The suggested module framework can be very valuable, especially considering it leaves options for customization.
I personally think that the literature review is quite complete, and the information reported give the reader a good picture of the topic.
I have a few suggestions that I hope may help making the paper even clearer and more useful to the reader. For example, I think the structure of the paper should match the structure of the figure, with a clear subdivision in modules and submodules (i.e., chapters and subchapters). While it surely is not the ideal behaviour, we are all aware that many readers will probably skim through the article to the different modules they are interested on. A more defined chapter structure of the article will make it more useful to a wider audience.
The writing is clear, and the article is well-written. I hope the authors will forgive me if I spent a bit of time making (probably picky) changes to the wording, especially in the conclusion. This is only because I think the paper really has value and the conclusion will be one of the most-read parts of the article once published.
I have a few main comments and some minor changes (below), but I think the article should be accepted after their corrections.
Please, add continuous line numbers when resubmitting.
Main comments:
Module 1: Sample acquisition.
- At page 7, the authors start with the "Sample acquisition" chapter, which is their "Module 1" in the figure. The title of this chapter should be "Module 1: Sample acquisition". Here the reader, after a first introductory paragraph where a sample is defined (see next comment), should be able to find three subsections: submodule 1.1: malaise traps, submodule 1.2: pitfall traps. This proposed structure could also help readers focus on the part they are interested in. For example, if a researcher is using only pitfall traps, they will go directly to submodule 1.2. What about submodule 1.3? It appears in the figure but not in the text. If the figure is just an example of submodules that can be added, the authors should state that.
- This module start saying that it's important to have a "sample definition", and I totally agree. However,

the authors do not provide one. It is true that a sample definition is strongly linked to the collection technique, but I think that we still require a sample definition, and I think the authors should be able to provide one. In my opinion, all the information necessary for a definition is in the text, it just need to be summarised. For example, a sample is composed by the arthropods, but also by their preservation, and the associated metadata. If any of these factors is missing, the sample is not fit for metabarcoding. It would be useful to know what else is really required for a sample to be defined as such. This sample definition should be after the first sentence and prior to the second one.

- After the first introductory aspects on definition of a sample, the authors cite the work of Montgomery and colleagues, where seven different collection methods are listed. The authors state that these methods provide "an appropriate platform from which to develop sample acquisition submodules". After reading this, I would have expected the authors to provide a submodule on EACH of these seven methods. Instead, only malaise traps and pitfall traps are presented. By doing this, the authors are either contradicting themselves and the work of Montgomery, or they are not clear on the reason they decide to report only two methods. Are the authors suggesting that, of the seven sampling techniques proposed, only malaise traps and pitfall traps are good for metabarcoding? Or are you suggesting that these two alone can provide good-enough results? Or again that, while all seven techniques are good, the authors are explaining only two? (If so, why?). In any case, this should be explained in detail.
- In addition to this, I would separate the sampling techniques from the metadata collection, or it could get very repetitive. In fact, independently of the collection method, the metadata information should always accompany the arthropods sample. For example, why in table 1.2 is not reported "Extreme weather events during trapping"? This is very important for pitfall traps, too. Indeed, a major rainfall could dilute the preservative or even make the trap overflow (with relative risk of losing specimens). While a very hot weather is known to cause evaporation, with the risk of drying the trap. The authors mention this in the text, but not in the table.
- In the same table, since it is reported the solution % for ethanol, also the glycol solution % should be reported. When using glycol in pitfall traps, the percentage should be lower than 95% (ideally between 40%/80% due to the viscosity of this preservative). At a 95% concentration, glycol may be so viscous that insect are not entirely submerged when they fall in the trap.

Module 2: Sample processing.

- As for the previous module, submodule paragraphs would be very helpful.
- I think the authors make an interesting point on the fact that size-sorting is not as necessary as one would think when deeper sequencing depth is an option. However, I have some issues with the explanations for this statement. The authors state that "increasing sequencing depth by 3-4 fold" to a "sufficient sequencing depth", together with "reasonable size ranges" make size-sorting superfluous. All these terms, unfortunately, are extremely subjective and do not enable the reader to understand when a sufficient sequencing depth is reached. Telling the reader that they need a "sufficient sequencing depth" to be able to ignore size-sorting is a tautology: it is obvious that if the sequencing depth is sufficient your work is good. In my opinion, the question readers would ask themselves is: what is a good sequencing depth in order for me to avoid size-sorting as the authors suggest? It is mentioned an increase of 3-4 fold, but that is relative to the whatever number of reads you had to start with.

It would probably be useful for the reader to understand what platform the authors are referring to at this stage, but that would also require the authors to explain how many samples they would process per

run. Depending on the work conducted, an increase of 3-4 folds in sequencing depth may mean the operator has to move from a MiSeq to a NovaSeq, for example. Or reduce the number of samples processed on each run (or their replicates). These factors should be considered, or at least mentioned, when suggesting that a higher sequencing depth is better than size-sorting.

If the reader makes it to the end of the modules, they will notice this topic is mentioned at page 21. I think, however, that the correlation between sample processing and sequencing depth is extremely important and should be explained in this module.

I agree that size-sorting is terribly time-consuming and therefore expensive; however, having to run your samples on two runs instead of one to get a better sequencing depth would be probably more expensive.

I am not sure if it can be useful to the authors, but Piper and colleagues (GigaScience, 8, 2019, 1-22, doi: 10.1093/gigascience/giz092, which is cited as reference number 7) provide a table with the costs and Gb output for each platform. This may be useful to give a reader an idea of what a good sequencing depth can be. Or link the readers to the page 21 explanation of the average reads-per-specimen expected in each sample.

Otherwise, a possibly simpler solution could be to provide the reader with a method to determine what a good sequencing depth looks like. For example, a taxa recovery graph that reaches plateau has been considered a valid and easy test to determine this (Hajibabaei et al. 2019 - PLoS One. 2019; 14(9): e0220953. doi: 10.1371/journal.pone.0220953).

Minor changes:

Page 5, line 4: remove "are". It should read: "by placing different fractions of terrestrial diversity at the core of each "module".

Page 6, first 8 lines of the "Harmonisation for the metabarcoding of terrestrial arthropods" paragraph: Compared to the rest of the introduction, this paragraph could be improved both in form and in content. It seems a few different topics have just been put together, with an isolated sentence for each, without going in depth enough and without linking the sentences to each other. I suggest the authors either rewrite this paragraph or simply list the reasons why arthropods assessment is useful (e.g., biodiversity assessment, conservation of declining species, monitoring of invasives). As per the form, the use of terms such as "overwhelming" and "tremendous" could be avoided (a bit too subjective), as it should be the repetition of the word "present" at line 2.

Page 6, last line: remove "in". It should read: "comparable to standard methods of arthropod monitoring".

Page 7, line 5: Close parenthesis after the references and remove the comma.

Page 7, first line of "Sample acquisition": "Starting point" instead of "departure point".

Page 15, "DNA extraction" Chapter, line 10: The authors mention the "taxonomic content of samples" and in bracket give the definition of OTUs. This can be confusing for the reader. The taxonomic content of a sample is not necessarily defined by OTUs, but could be extrapolated using ASVs (amplicon sequence variants). Since the authors are referring to a specific paper they are referencing, I suggest to change the sentence to: "When assessing the recovered taxonomic content of samples using operational taxonomic units (OTUs), intact samples performed at least comparable.

Page 16: The authors suggest that 100-200 Î¼l of DNA extraction buffer can be considered appropriate for harmonisation. This gives the impression the authors are suggesting to use only 200 units of buffer

when performing the DNA extraction. In my experience, an average pitfall trap that has been in the field for a week an contains even just 2 bees and 2 beetles (very unlikely) can easily require almost 1 ml of buffer when using a non-destructive DNA extraction method. As the authors stated a few sentences earlier, this is a large volume of buffer. Then why suggesting that 100-200 Î¼l is enough? Was this referring to the use of just 100-200 Î¼l as a subsample to purify from the overall volume used? If so, the sentence should read something like:
"Given this consideration, typical commercial kit extraction volumes of 100-200 Î¼l can be considered an appropriate sub-sampling volume for subsequent purification."
Page 17, Chapter 4: gene names should be italicised. Correct to: "Cytochrome c oxidase subunit I barcode region". Please, note that "subunit I" is not part of the name and should not be italicised.
Page 18, Line 3: I would break the sentence in two: "The BF3 fragment (418 bp) provides better taxonomic resolution than other overlapping fragments. Furthermore, primers within this region are also unaffected by slippage, and provide maximum overlap across already published studies."
Page 18, Line 18: Reference is missing, check "ref".
Page 18, Line 20: My understanding is that the proofreading activity of a polymerase is the 3â€²â†’5â€² exonuclease activity. I am not sure what the "non" refers to. I think it should read: "their proofreading activity (3â€²â†’5â€² exonuclease activity)"
Page 23, "Conclusion" Line 1: No need to give both the full name and the abbreviation for wocDNA, since this was done previously. The authors can pick one.
Page 23, "Conclusion" Line 7: "address this issue".
Page 23, "Conclusion" Line 8 and 11: the use of the term "canalization", while technically correct, seems a bit odd and adds unnecessary jargon, especially considering the conclusion will be read by most readers. I would suggest changing this term.
Page 23, "Conclusion" Line 13 and 14: "submodule", "modular" and "modules" in the same sentence makes it very hard to read.. A possible solution could be:
"the flexible structure we presented here seeks to broaden the applicability of a modular framework within the wocDNA metabarcoding community."
Page 23, "Conclusion" Line 18: Again, it is a bit repetitive to mention the submodule structure of the module. If it is a submodule, then it is already given that is part of the module. I would rephrase by removing "module".

Methods

Are the methods appropriate to the aims of the study, are they well described, and are necessary controls included? Choose an item.

Conclusions

Are the conclusions adequately supported by the data shown? Choose an item.

Reporting Standards

Does the manuscript adhere to the journal's guidelines on [minimum standards of reporting?]() Choose an item.

Choose an item.

Statistics

Are you able to assess all statistics in the manuscript, including the appropriateness of statistical tests used? Choose an item.

Quality of Written English

Please indicate the quality of language in the manuscript: Choose an item.

Declaration of Competing Interests

Please complete a declaration of competing interests, considering the following questions:

- Have you in the past five years received reimbursements, fees, funding, or salary from an organisation that may in any way gain or lose financially from the publication of this manuscript, either now or in the future?
- Do you hold any stocks or shares in an organisation that may in any way gain or lose financially from the publication of this manuscript, either now or in the future?
- Do you hold or are you currently applying for any patents relating to the content of the manuscript?
- Have you received reimbursements, fees, funding, or salary from an organization that holds or has applied for patents relating to the content of the manuscript?
- Do you have any other financial competing interests?
- Do you have any non-financial competing interests in relation to this paper?

If you can answer no to all of the above, write 'I declare that I have no competing interests' below. If your reply is yes to any, please give details below.

I declare that I have no competing interests.

I agree to the open peer review policy of the journal. I understand that my name will be included on my report to the authors and, if the manuscript is accepted for publication, my named report including any attachments I upload will be posted on the website along with the authors' responses. I agree for my report to be made available under an Open Access Creative Commons CC-BY license (http://creativecommons.org/licenses/by/4.0/). I understand that any comments which I do not wish to be included in my named report can be included as confidential comments to the editors, which will not be published.

Choose an item.

To further support our reviewers, we have joined with Publons, where you can gain additional credit to further highlight your hard work (see: https://publons.com/journal/530/gigascience). On publication of this paper, your review will be automatically added to Publons, you can then choose whether or not to claim your Publons credit. I understand this statement.

Yes Choose an item.
