# Supplementary material for: Toward global integration of biodiversity big data: a harmonized metabarcode data generation module for terrestrial arthropods
Source: Gigascience. 2022 Jul 19;11:giac065. doi: 10.1093/gigascience/giac065 (PMC9295367; doi:10.1093/gigascience/giac065)
Supplement: giac065_Reviewer_2_Report_Original_Submission — Andrew Dopheide -- 2/7/2022 Reviewed [file giac065_reviewer_2_report_original_submission.pdf]

Reviewer Report

Title: Toward global integration of biodiversity big data: a harmonised metabarcode data generation module for terrestrial arthropods

Version: Original Submission **Date:** 2/7/2022

Reviewer name: Andrew Dopheide

Reviewer Comments to Author:

The manuscript makes a well-argued case for the adoption of consistent metabarcoding data generation workflows (harmonisation) for inventorying macro-biodiversity, within a modular framework, to enable larger-scale analyses that incorporate multiple datasets - and this is clearly a good idea. To do this, the authors review the relevant literature, and based on this, provide sets of workflow recommendations, at five key data generation steps, within a proposed terrestrial arthropod metabarcoding module.
The paper is largely well written and easy to follow (apart from some parts detailed in the line-by-line comments below). The authors have done an excellent job of reviewing the relevant literature, and the manuscript is packed with useful workflow recommendations for metabarcoding of terrestrial invertebrates. A particularly helpful aspect is the consideration of all data generation steps, from initial sampling through to the storage of sequence data and metadata.
One possible omission is that almost no mention is made of arthropods living below ground, which is an important component of terrestrial arthropod biodiversity, with another set of sampling methods and considerations. Given that the manuscript focuses on workflows for "terrestrial arthropods", I think it should at least be mentioned that that sampling for soil arthropod metabarcoding would be another submodule, but is not considered in this manuscript. Similarly, it might be helpful to suggest other modules that could or should be developed, within the conclusion?
Are these modules going to exist anywhere apart from within this manuscript and subsequent manuscripts? It might be helpful to have a website that collects all these modules into one place for easy access, somewhat like the Earth microbiome
project website.
L 34: For inventorying biodiversity? For compiling biodiversity inventories?
L 79: It is unclear whether "metabarcode inventory data" means the data resulting from metabarcoding analyses, or the data about metabarcoding methods/workflows?
L 89: I think "global microbial initiatives" is missing something. Global microbial diversity assessment initiatives? Also, I'm not sure "(even if data generation has been centralised)" is needed.
L 94: What are eDNA initiatives, as opposed to metabarcoding initiatives?
L 98: "one of the most heterogeneous groups in terms of body size"?
L 99: I think it would be clearer to use "inventorying of" (i.e. compiling an inventory), rather than "inventory". (Inventorying is used elsewhere, e.g. L 108, 166).
L 110: "calibration and so" seems unnecessary.
L 111: It's unclear to me why catalysis of a GO network is the key challenge. Perhaps consistent workflows are implicit in a GO network? But consistent workflows could exist without a GO network too.

Can you clarify how a GO network helps?
L 119-122: Arguably, bioinformatic processing of raw sequence data into processed data is another key step (depending on whether "data" is the raw sequence data, or processed OTU/ASV data). Evidently, this is not within the scope of the manuscript, but it might be worth mentioning somewhere that post-sequencing aspects of metabarcoding workflows can also vary a lot, resulting in incomparable datasets. However, this is less problematic because one can theoretically re-process the sequence data from different studies in a consistent manner.
L 140-142: This sentence is very confusing. "long-view" should probably be "long-term goal"; "synthetic analyses" sounds like analyses of synthetic (artificial or man-made) data; and I'm not sure what "a function of any collateral costs" means. Please rephrase.
L 144: minimal compromise, if any?
L 150: The declines of insects (plural) are now a very real and serious threat?
L 161: inventorying arthropod biodiversity?
L 162: Remove "in".
L 183-184: panacea? Might be better to say "no one method detecting the entire arthropod diversity within a site"
L 273: I'm not sure "for harmonisation" is needed here.
L 321: Photographing of invertebrate samples is an excellent idea!
L 330: Would there be a benefit to trying to orient all the specimens in the same way, for potential future visual-based identifications? (probably time-consuming though).
L 307: "4mm sieve pooled 1:10 to 2:10" is unclear. Does it mean, the < 4mm and > 4mm fractions are pooled together at a ratio of 1:10 to 2:10? Which fraction is the higher ratio? Please clarify.
L 337: What is a SuperGO?
L 398-405: "COI-bcr" is unnecessary, only used in this paragraph. "COI barcode" is used on line 408 to mean the same thing, and is clearer. I suggest replacing "COI-bcr" on lines 401 and 405 with "COI barcode" and COI barcode region",
respectively.
L 405-406: This sentence should be rephrased. Multiple COI-targeted primer sets … demonstrated to efficiently characterise arthropods … particularly those with certain degenerate positions?
L 407: see Figure 2 in Elbrecht et al.? Should "second half" be 3' (prime)?
L 408-412: Can you provide citation for claims about BF3, and for primers BF2, III_B_F, Fol-degen-rev? I think the "primers within this region…" statement should be qualified with a word such as "published" or "tested". Maximum overlap of what among already published studies? (COI regions?) Do these primers have any limitations in terms of taxonomic coverage?
L 412: Why "eDNA metabarcoding" here, but just "metabarcoding" everywhere else?
L 424: Citation missing?
L 471: Why would that be so? (Lower cost?)
Table 5: What is BC3 fragment? I'm not sure how useful it is to recommend "degenerate primers" here - presumably it is certain specific COI-targeted degenerate primers that are recommended, not degenerate primers in general, in which case should they be listed here?
L 533: I'm not sure what canalisation means. Replace with harmonisation?

L 539-540: "of modules" seems repetitive (of submodules) and unnecessary.

Methods

Are the methods appropriate to the aims of the study, are they well described, and are necessary controls included? Choose an item.

Conclusions

Are the conclusions adequately supported by the data shown? Choose an item.

Reporting Standards

Does the manuscript adhere to the journal's guidelines on minimum standards of reporting? Choose an item.

Choose an item.

Statistics

Are you able to assess all statistics in the manuscript, including the appropriateness of statistical tests used? Choose an item.

Quality of Written English

Please indicate the quality of language in the manuscript: Choose an item.

Declaration of Competing Interests

Please complete a declaration of competing interests, considering the following questions:

- Have you in the past five years received reimbursements, fees, funding, or salary from an organisation that may in any way gain or lose financially from the publication of this manuscript, either now or in the future?
- Do you hold any stocks or shares in an organisation that may in any way gain or lose financially from the publication of this manuscript, either now or in the future?
- Do you hold or are you currently applying for any patents relating to the content of the manuscript?
- Have you received reimbursements, fees, funding, or salary from an organization that holds or has applied for patents relating to the content of the manuscript?
- Do you have any other financial competing interests?
- Do you have any non-financial competing interests in relation to this paper?

If you can answer no to all of the above, write 'I declare that I have no competing interests' below. If your reply is yes to any, please give details below.

I declare that I have no competing interests

I agree to the open peer review policy of the journal. I understand that my name will be included on my report to the authors and, if the manuscript is accepted for publication, my named report including any attachments I upload will be posted on the website along with the authors' responses. I agree for my report to be made available under an Open Access Creative Commons CC-BY license (http://creativecommons.org/licenses/by/4.0/). I understand that any comments which I do not wish to be included in my named report can be included as confidential comments to the editors, which will not be published.

Choose an item.

To further support our reviewers, we have joined with Publons, where you can gain additional credit to further highlight your hard work (see: https://publons.com/journal/530/gigascience). On publication of this paper, your review will be automatically added to Publons, you can then choose whether or not to claim your Publons credit. I understand this statement.

Yes Choose an item.
